# Tel1 and Rif2 Regulate MRX Functions in End-Tethering and Repair of DNA Double-Strand Breaks

**DOI:** 10.1371/journal.pbio.1002387

**Published:** 2016-02-22

**Authors:** Corinne Cassani, Elisa Gobbini, Weibin Wang, Hengyao Niu, Michela Clerici, Patrick Sung, Maria Pia Longhese

**Affiliations:** 1 Dipartimento di Biotecnologie e Bioscienze, Università di Milano-Bicocca, Milano, Italy; 2 Department of Molecular Biophysics and Biochemistry, Yale University School of Medicine, New Haven, Connecticut, United States of America; 3 Department of Molecular and Cellular Biochemistry, Indiana University, Bloomington, Indiana, United States of America; Mount Sinai Hospital, CANADA

## Abstract

The cellular response to DNA double-strand breaks (DSBs) is initiated by the MRX/MRN complex (Mre11-Rad50-Xrs2 in yeast; Mre11-Rad50-Nbs1 in mammals), which recruits the checkpoint kinase Tel1/ATM to DSBs. In *Saccharomyces cerevisiae*, the role of Tel1 at DSBs remains enigmatic, as *tel1*Δ cells do not show obvious hypersensitivity to DSB-inducing agents. By performing a synthetic phenotype screen, we isolated a *rad50-V1269M* allele that sensitizes *tel1*Δ cells to genotoxic agents. The MR^V1269M^X complex associates poorly to DNA ends, and its retention at DSBs is further reduced by the lack of Tel1. As a consequence, *tel1*Δ *rad50-V1269M* cells are severely defective both in keeping the DSB ends tethered to each other and in repairing a DSB by either homologous recombination (HR) or nonhomologous end joining (NHEJ). These data indicate that Tel1 promotes MRX retention to DSBs and this function is important to allow proper MRX-DNA binding that is needed for end-tethering and DSB repair. The role of Tel1 in promoting MRX accumulation to DSBs is counteracted by Rif2, which is recruited to DSBs. We also found that Rif2 enhances ATP hydrolysis by MRX and attenuates MRX function in end-tethering, suggesting that Rif2 can regulate MRX activity at DSBs by modulating ATP-dependent conformational changes of Rad50.

## Abstract 2

This study reveals novel roles for the checkpoint kinase Tel1/ATM and Rif2 in regulating the function of the MRX complex during repair of DNA double-strand breaks by nonhomologous end joining and homologous recombination.

## Author Summary

Many tumors contain mutations that confer defects in repairing DNA double-strand breaks (DSBs). In both yeast and mammals, the MRX/MRN complex (Mre11-Rad50-Xrs2 in yeast; Mre11-Rad50-Nbs1 in mammals) plays critical functions in repairing a DSB by either nonhomologous end joining (NHEJ) or homologous recombination (HR). Furthermore, it recruits the checkpoint kinase Tel1/ATM. Although ATM is considered to be a tumor suppressor, up-regulation of ATM signaling promotes chemoresistance, radioresistance and metastasis. For this reason, cancer therapies targeting ATM have been developed to increase the effectiveness of standard genotoxic treatments and/or to set up synthetic lethal approaches in cancers with DNA repair defects. We aimed to identify the precise role of ATM/Tel1 in these processes. By performing a synthetic phenotype screen, we identified a mutation (*rad50-V1269M*) altering the Rad50 subunit of the MRX complex, which sensitizes cells lacking Tel1 to genotoxic agents. Genetic and biochemical characterization of MR^V1269M^X protein complex revealed that Tel1 promotes MRX association at DSBs to allow proper MRX-DNA binding that is needed for DSB repair. The role of Tel1 in promoting MRX retention on DSBs is counteracted by Rif2, which can regulate MRX activity at DSBs by modulating ATP-dependent conformational changes in Rad50. Our finding that MRX dysfunctions can be synthetically lethal with Tel1 loss in the presence of genotoxic agents suggests that ATM inhibitors could be beneficial in patients whose tumors have defective MRN functions.

## Introduction

DNA double-strand breaks (DSBs) are among the most cytotoxic DNA lesions, because failure to repair them can lead to genome instability. DSBs can be repaired by either nonhomologous end joining (NHEJ) or homologous recombination (HR). While NHEJ directly ligates the DNA ends, HR requires the 5′ ends of a DSB to be nucleolytically processed (resected) to generate 3′ single-stranded DNA (ssDNA) tails that initiate HR by invading an undamaged homologous DNA template [1].

Generation of DSBs activates a DNA damage response (DDR), which regulates DSB repair and coordinates it with cell cycle progression [2]. In both yeast and mammals, the DDR is initiated by the MRX/MRN complex (Mre11-Rad50-Xrs2 in yeast; Mre11-Rad50-Nbs1 in mammals), which recognizes unprocessed DSBs and activates the checkpoint kinase Tel1/ATM [3]. MRX/MRN recruits Tel1/ATM to the DSB ends through its interaction with the C-terminal domain of Xrs2/Nbs1 and stimulates Tel1/ATM catalytic activity [4–8].

MRX/MRN also plays critical functions in DSB resection and in maintaining the DSB ends tethered to each other [9]. Several studies have shown that the MRX complex consists of a globular head domain from which the long coiled-coil domain of Rad50 protrudes [10–13]. The coiled-coil apex contains a CXXC amino acid motif that can dimerize via tetrahedral coordination of a zinc ion, thereby forming molecular bridges for keeping the DNA ends tethered to each other [14,15]. Mre11 is active as an exo- and endonuclease in vitro [16–19] and initiates DSB resection [20–24]. The functions of MRX in end-tethering and DSB resection are regulated by Rad50, whose ATP binding and hydrolysis activities result in MRX conformational changes [11,25–28]. Mutants that promote the ATP-bound conformation of Rad50 exhibit a higher level of tethering [29], indicating that end-tethering depends on this MRX conformation. In turn, the ATP-bound conformation sterically blocks the Mre11 nuclease activity [29–32], whereas release from this ATP-bound state that occurs with ATP hydrolysis opens Mre11 nuclease active sites so that they can be engaged in DSB resection [13]. Thus, ATP hydrolysis triggers a switch between a closed state, in which Mre11 nuclease domain is occluded, to an open configuration with exposed Mre11 nuclease sites.

In addition to its role in DSB repair, MRX works in the same epistasis group of Tel1 to maintain telomere length [33,34]. Interestingly, the lack of Tel1 in *Saccharomyces cerevisiae* cells causes telomere shortening and a decrease of MRX binding at DNA ends flanked by telomeric DNA repeats [35,36]. On the other hand, telomere length is negatively regulated by Rif2, which is recruited to telomeric DNA ends by Rap1 [37]. Artificial tethering of Rif2 at DNA ends reduces the amount of telomere-bound Tel1, but not that of MRX [35]. This observation, together with the finding that Rif2 appears to compete with Tel1 for binding to the C-terminus of Xrs2 in vitro [35], suggests that Rif2 interferes with MRX-Tel1 interaction to shelter telomeric ends from Tel1 recognition.

Although Tel1 is recruited to DSBs and participates in DSB end resection [4,38], its function in DSB repair remains enigmatic because Tel1-deficient *S*. *cerevisiae* cells do not show obvious hypersensitivity to DNA damaging agents and are not defective in checkpoint activation in response to a single DSB [38]. To better understand the function of Tel1 in the cellular response to DSBs, we performed a genetic screen aimed at identifying mutants that require Tel1 to survive to genotoxic treatments. We found that the *rad50-V1269M* allele makes *tel1*Δ cells hypersensitive to DNA damaging agents. The MR^V1269M^X complex associates poorly to a DSB and the lack of Tel1 further reduces its retention at DSB ends. As a consequence, *rad50-V1269M tel1*Δ cells are severely defective in maintaining the DSB ends tethered to each other. These findings indicate that Tel1 promotes proper MRX association to DNA ends, and this function is required to support the end-tethering activity of MRX. The Tel1 function in promoting MRX retention to DSBs is counteracted by Rif2, which is recruited to DSB ends. Rif2 also enhances MRX ATPase activity and attenuates MRX function in end-tethering, suggesting that it modulates MRX function not only by inhibiting MRX association to DSBs but also by regulating ATP-dependent Rad50 conformational changes.

## Results

### *rad50-V1269M* Cells Require Tel1 for DNA Damage Resistance

**Fig 1:**
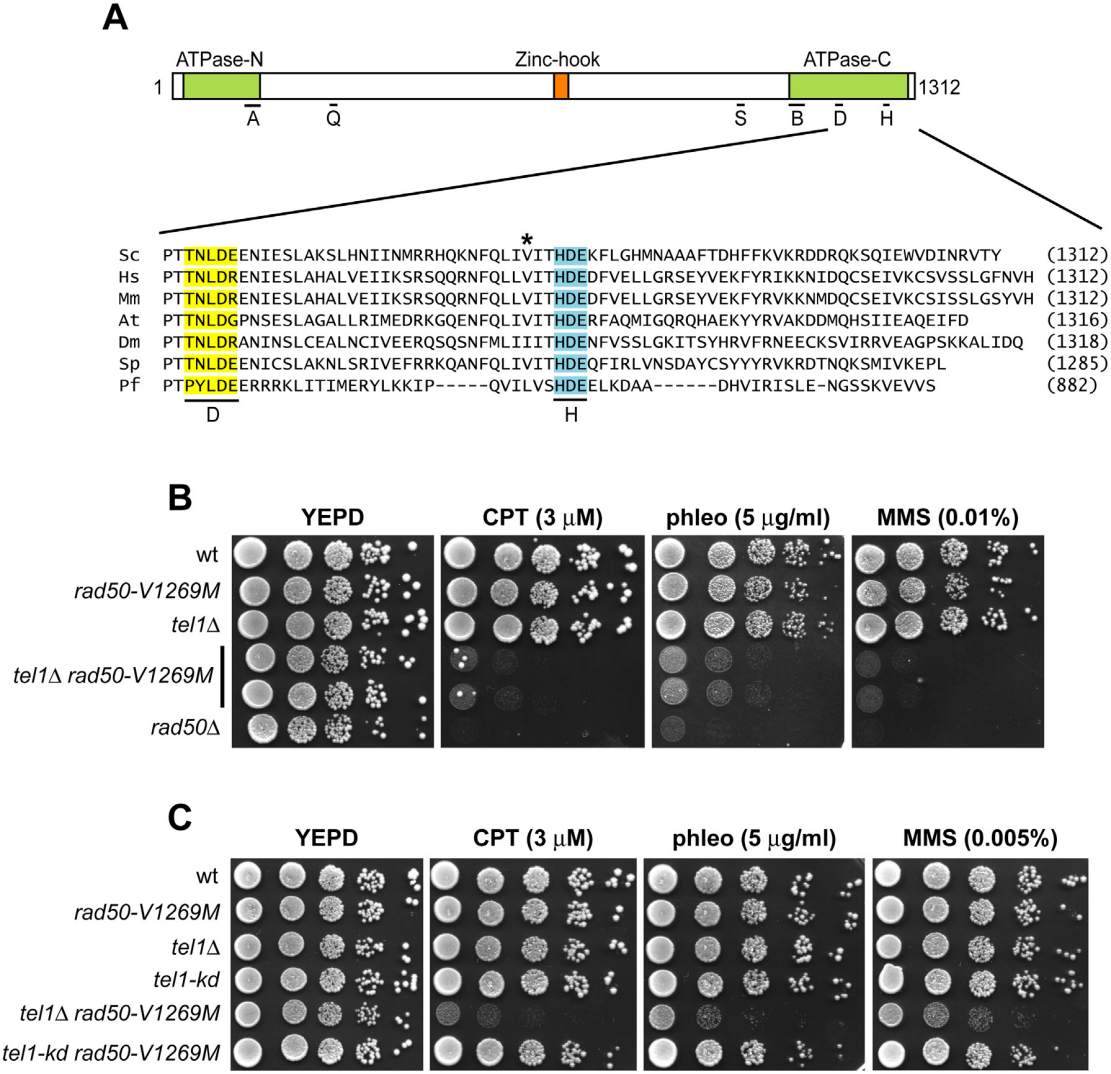
The *rad50-V1269M* mutation sensitizes *tel1*Δ cells to DNA damaging agents. (A) Multiple sequence alignment across seven organisms of Rad50 D-loop (yellow), H-loop (blue) and adjacent sequences. The valine residue substituted by methionine in Rad50-V1269M mutant is indicated by an asterisk. Abbreviations: A, Walker A; Q, Q-loop; S, Signature; B, Walker B; D, D-loop; H, H-loop; *Sc*, *Saccharomyces cerevisiae*; *Hs*, *Homo sapiens*; *Mm*, *Mus musculus*; *At*, *Arabidopsis thaliana*; *Dm*, *Drosophila melanogaster*; *Sp*, *Schizosaccharomyces pombe*; *Pf*, *Pyrococcus furiosus*. (B, C) Exponentially growing cultures were serially diluted (1:10) and each dilution was spotted out onto yeast extract peptone dextrose (YEPD) plates with or without CPT, phleomycin or methyl methanesulfonate (MMS) at the indicated concentrations.

To gain insights into the role of Tel1 at DSBs, we searched for mutations that caused hypersensitivity to DNA damaging agents only in the absence of Tel1. For this purpose, *tel1*Δ clones were screened for decreased viability in the presence of camptothecin (CPT) and/or phleomycin. Hypersensitive *tel1*Δ clones that lost the DNA damage hypersensitivity after transformation with a plasmid containing wild-type *TEL1* were crossed to a wild-type strain followed by sporulation and tetrad analysis to verify that the DNA damage hypersensitivity was due to the combination of *tel1*Δ with a mutation in an unknown single gene. This procedure allowed us to identify five single-gene mutations belonging to three distinct allelism groups. Genome sequencing of the clone that showed the most severe synthetic phenotype and subsequent genetic analyses established that the mutation responsible for the DNA damage hypersensitivity of *tel1*Δ cells was a single nucleotide change in the *RAD50* gene, resulting in substitution of valine 1269 with methionine in the C-terminal ATPase domain (Fig 1A).

Both *rad50-V1269M* and *tel1*Δ single mutant cells were as sensitive as wild type to phleomycin, methyl methanesulfonate (MMS), and low CPT doses, while the sensitivity to the same drugs was greatly increased in *tel1*Δ *rad50-V1269M* double mutant cells (Fig 1B), indicating that the Rad50-V1269M variant requires Tel1 to support cell viability in the presence of genotoxic stress.

As Tel1 is a protein kinase, we asked whether the *rad50-V1269M* allele also exacerbated the sensitivity to DNA damaging agents of cells expressing a Tel1 mutant variant (Tel1-kd) carrying G2611D, D2612A, N2616K, and D2631E amino acid substitutions that abolished Tel1 kinase activity in vitro [39]. Telomeres in *tel1-kd* cells are shorter than in wild-type cells and indistinguishable from those of *tel1*Δ cells [39], indicating that these mutations abolish Tel1 function at telomeres. Surprisingly, the viability of *tel1-kd rad50-V1269M* double mutant cells in the presence of DNA damaging agents was similar to wild-type cells (Fig 1C), suggesting that Rad50-V1269M mutant variant requires the presence of Tel1 but not its kinase activity to support cell viability in the presence of genotoxic stress.

### Rad50-V1269M Exhibits Reduced DNA Binding and ATP Hydrolysis

**Fig 2:**
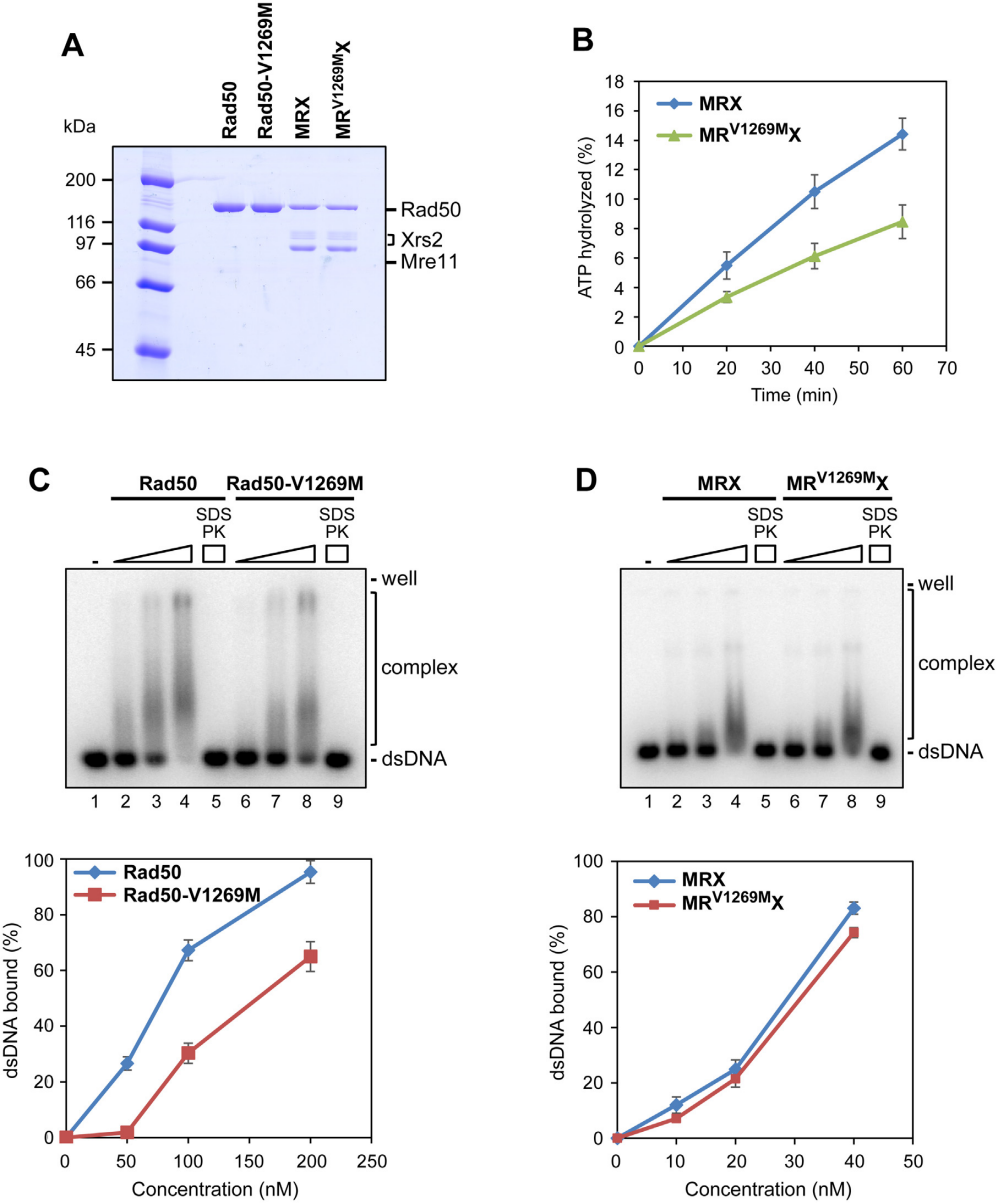
The *rad50-V1269M* mutation impairs Rad50 ATPase and binding to DNA in vitro. (A) Purified Rad50, Rad50-V1269M, MRX, and MR^V1269M^X, 1 μg each, were analyzed by SDS-PAGE and stained with Coomassie Blue. (B) The ATPase activity of wild-type MRX and mutant MR^V1269M^X complexes was determined as described in [27]. Plotted values are the mean value with error bars denoting standard deviation (s.d.) (*n* = 3). (C) Rad50 and Rad50-V1269M proteins (50, 100, and 200 nM) were incubated with ^32^P-labeled DNA (10 nM) in the presence of ATP. In lane 5 and 9, the reaction mixture was deproteinized with SDS and proteinase K (PK) prior to analysis. Plotted values are the mean value with error bars denoting s.d. (*n* = 3). (D) MRX and MR^V1269M^X complexes (10, 20, and 40 nM) were treated as in (C).

Rad50 binds DNA and has ATPase activity [27]. These functions reside in the globular domain formed by the N- and C-termini of the protein, which are separated by an antiparallel coiled-coil domain [9]. The V1269M mutation is very closed to the H-loop (Fig 1A), whose histidine residue has been proposed to promote ATP hydrolysis by positioning the first water molecule needed for the reaction and/or by forming a catalytic dyad with the Walker B glutamate [11,40]. Thus, we asked whether and how the *rad50-V1269M* mutation affects MRX ATPase and/or DNA-binding activities. The Rad50 and the Rad50-V1269M proteins were purified to near homogeneity by following our published procedure (Fig 2A) [18]. Purified Rad50 and Rad50-V1269M were then individually incubated with Mre11 and Xrs2, and the fully assembled complexes were separated from free proteins by gel filtration. Rad50-V1269M could be expressed to the same level as the wild-type protein, behaved well chromatographically, and yielded the same amount of trimeric complex with Mre11 and Xrs2. As shown in Fig 2A, the stoichiometry of the three components in the MR^V1269M^X mutant complex was very similar to that of the wild-type MRX complex.

As we reported previously [41], Rad50 hydrolyzed ATP only within the context of the MRX complex. The MR^V1269M^X mutant complex exhibited a reduced ATPase activity compared to wild-type MRX (Fig 2B), indicating that the *rad50-V1269M* mutation affects ATP hydrolysis.

Aside from Xrs2 and Mre11, Rad50 also binds DNA [41], and we found that Rad50-V1269M is compromised for DNA binding in vitro (Fig 2C). Subsequently, we examined DNA binding by MRX wild-type and MR^V1269M^X mutant complexes and noticed insignificant difference between the two complexes (Fig 2D). We note that the DNA binding deficiency of the Rad50-V1269M mutant may be masked by the DNA binding attribute of Mre11 and Xrs2 within the MRX complex [19,41].

**Fig 3:**
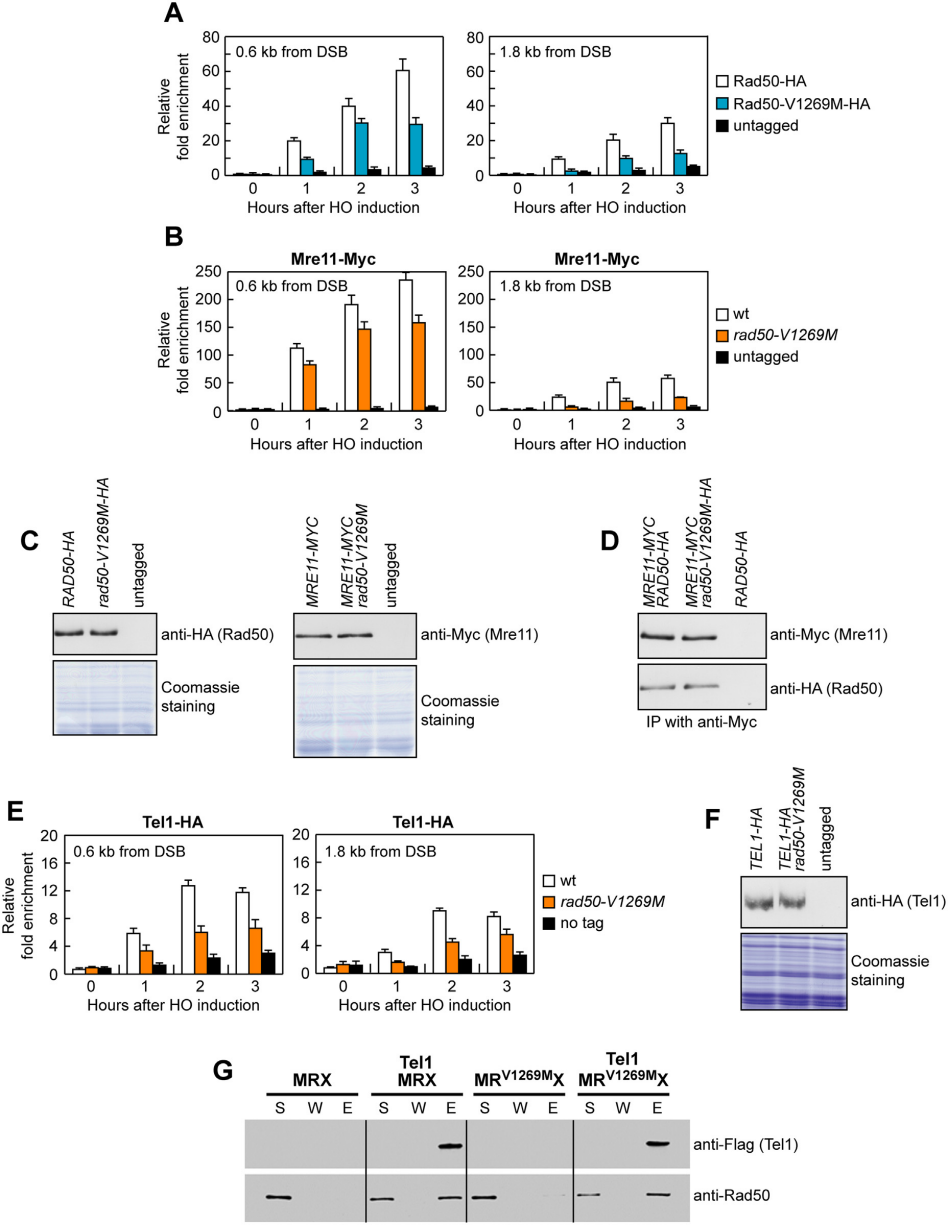
The *rad50-V1269M* allele impairs MRX association to a DSB in vivo. (A, B) ChIP analysis. Exponentially growing YEPR cell cultures were transferred to YEPRG at time zero. Relative fold enrichment of the indicated fusion proteins at the indicated distances from the HO cleavage site was determined after ChIP with anti-HA or anti-Myc antibodies and subsequent qPCR analysis. Plotted values are the mean value with error bars denoting s.d. (*n* = 3). (C) Western blot with anti-HA and anti-Myc antibodies of extracts used for the ChIP analysis shown in (A) and (B), respectively. The same amount of extracts was separated on a SDS-PAGE and stained with Coomassie Blue as loading control. (D) Mre11-Myc was immunoprecipitated with anti-Myc antibodies and the immunoprecipitates were subjected to western blot analysis using anti-HA or anti-Myc antibodies. (E) As in (A), but showing Tel1-HA association to the DSB. (F) Western blot with anti-HA antibodies of extracts used for the ChIP analysis shown in (E). (G) Flag-tagged Tel1 (50 ng) was incubated with wild-type MRX or MR^V1269M^X (100 ng of each) and protein complexes were captured by anti-Flag beads. The supernatant (S) containing unbound proteins, the wash (W), and the eluate (E) fractions were analyzed by immunoblotting.

We next analyzed DSB association of Rad50-V1269M and MR^V1269M^X in vivo by chromatin immunoprecipitation (ChIP) followed by quantitative real-time PCR (qPCR). To generate a single DSB at a specific chromosomal locus, we used a strain expressing a galactose-inducible HO endonuclease. In this strain, induction of HO by galactose addition leads to the generation at the *MAT* locus of a single DSB that cannot be repaired by HR because the strain carries the deletion of the homologous donor loci *HML*α and *HMR*a [42]. Consistent with the finding that the Rad50-V1269M mutant variant is compromised in DNA binding (Fig 2C), the amount of Rad50-V1269M bound at the HO-induced DSB was lower than that of wild-type Rad50 (Fig 3A). Furthermore, although binding to DNA of the MR^V1269M^X mutant complex was not affected (Fig 2D), the amount of Mre11 associated to the HO-induced DSB was significantly lower in *rad50-V1269M* than in wild-type cells (Fig 3B). This decreased Rad50-V1269M and MR^V1269M^X association to the DSB is not due to reduced protein levels or altered MR^V1269M^X complex formation. In fact, protein extracts from wild-type and *rad50-V1269M* cells contained very similar amounts of Rad50, Rad50-V1269M, and Mre11 proteins (Fig 3C). Furthermore, equal amount of Rad50-V1269M and Rad50 could be immunoprecipitated with the Mre11 protein (Fig 3D). As MR^V1269M^X binding to DNA was not significantly affected (Fig 2D), these data indicate that the *rad50-V1269M* mutation impairs MRX retention to DSBs.

As expected from the previous finding that MRX is required to load Tel1 at the DSB ends [4], the amount of Tel1 bound at the HO-induced DSB was lower in *rad50-V1269M* cells than in wild type (Fig 3E). This attenuated Tel1 association to the DSB is not due to either reduced Tel1 level in *rad50-V1269M* cells or impaired MR^V1269M^X-Tel1 interaction. In fact, similar Tel1 amounts could be detected in protein extracts prepared from wild-type and *rad50-V1269M* cells (Fig 3F). Furthermore, wild-type and mutant MRX complexes could be coimmunoprecipitated equally well with Tel1 (Fig 3G).

### The Lack of Tel1 Does Not Impair MR^V1269M^X Function in DSB Resection and Checkpoint Activation

**Fig 4:**
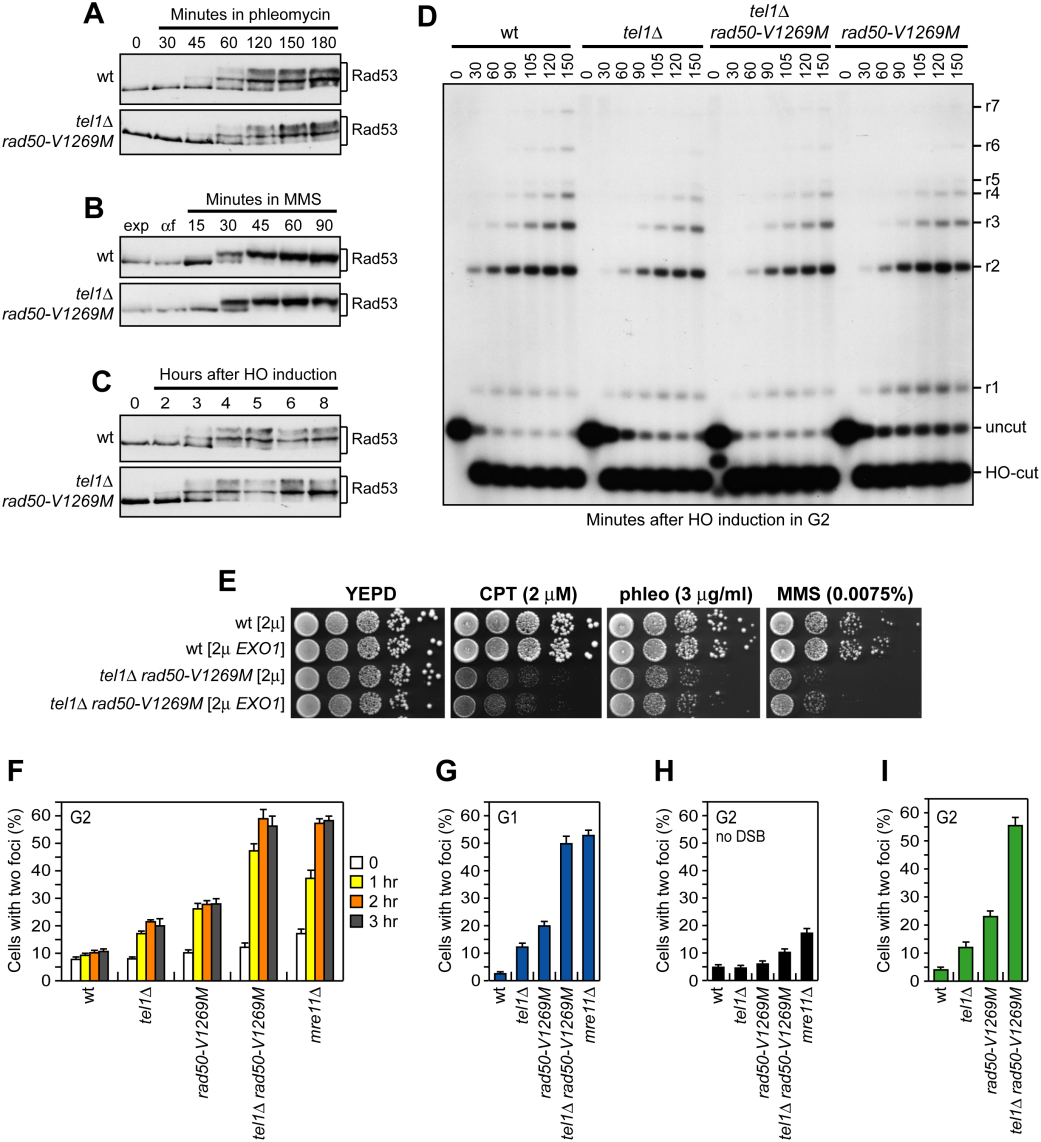
DSB resection and end-tethering. (A) Exponentially growing cell cultures were incubated with phleomycin (15 μg/ml) at time zero, followed by western blot analysis with anti-Rad53 antibodies. (B) Exponentially growing cell cultures (exp) were arrested in G1 with α-factor (αf) and then released in YEPD containing 0.015% MMS, followed by western blot analysis with anti-Rad53 antibodies. (C) Exponentially growing YEPR cell cultures were transferred to YEPRG at time zero to induce HO that catalyzes an irreparable DSB at the *MAT* locus. Protein extracts were analyzed by western blot analysis with anti-Rad53 antibodies. (D) DSB resection. YEPR exponentially growing cell cultures were arrested in G2 with nocodazole and transferred to YEPRG in the presence of nocodazole at time zero. SspI-digested genomic DNA separated on alkaline agarose gel was hybridized with a single-stranded *MAT* probe that anneals with the unresected strand. Resection of the 5′ DNA end progressively eliminates SspI sites, producing larger SspI fragments (r1 through r7) detected by the probe. (E) Exponentially growing cultures were serially diluted (1:10) and each dilution was spotted out onto YEPD plates with or without CPT, phleomycin, or MMS. (F, G) DSB end-tethering. Exponentially growing YEPR cell cultures were arrested in G2 with nocodazole (F) or in G1 with α-factor (G) at time zero and transferred to YEPRG in the presence of nocodazole or α-factor, respectively. Two-hundred cells for each strain were analyzed to determine the percentage of cells showing two LacI-GFP foci. (H) Sister chromatid cohesion. Exponentially growing YEPD cell cultures were arrested in G2 with nocodazole for 3 h to determine the percentage of cells showing two LacI-GFP foci. (I) Exponentially growing YEPR cell cultures, transformed with plasmid containing a galactose-inducible *HO* gene, were arrested in G2 with nocodazole at time zero and transferred to YEPRG in the presence of nocodazole for 2 h to determine the percentage of cells showing separated LacI-YFP and TetR-RFP foci. In all graphs, plotted values are the mean value with error bars denoting s.d. (*n* = 3).

The MRX complex plays multiple functions in DSB repair: it promotes DSB resection and checkpoint activation [20,21,38] and it keeps the DSB ends tethered to each other [43–46]. The severe DNA damage hypersensitivity of *tel1*Δ *rad50-V1269M* cells is not due to defect in DNA damage-induced checkpoint activation, as wild-type and *tel1*Δ *rad50-V1269M* cells phosphorylated the checkpoint kinase Rad53 with similar kinetics in response to phleomycin (Fig 4A) or MMS treatment (Fig 4B). Furthermore, *tel1*Δ *rad50-V1269M* cells phosphorylated Rad53 with wild-type kinetics in response to an irreparable HO-induced DSB (Fig 4C).

Recent data indicate that MRX in the ATP-bound state promotes end-tethering, whereas ATP hydrolysis opens the MRX conformation to promote Mre11 nuclease activity and DSB resection [13,29]. As MR^V1269M^X exhibits reduced ATPase activity and MRX variants impaired in ATP hydrolysis are endonuclease-defective [27,47], we asked whether *rad50-V1269M* cells were defective in DSB resection. To monitor directly the generation of ssDNA at the DSB ends, we used strains expressing a galactose-inducible HO endonuclease, which generates at the *MAT* locus a single irreparable DSB [42]. Resection of the HO-induced DSB renders the DNA sequence flanking the HO break resistant to cleavage by restriction enzymes, resulting in the appearance of resection intermediates that can be detected by Southern blot analysis with a probe that anneals to the 3′ end at one side of the break. Because resection was much slower in cells arrested in G2/M than in replicating cells [48], HO was induced by galactose addition to cell cultures that were arrested in G2 with nocodazole and kept blocked in G2 by nocodazole treatment to detect even subtle differences in resection efficiency. Consistent with MR^V1269M^X deficiencies in ATP hydrolysis and DNA binding, *rad50-V1269M* mutant cells showed a slight defect in DSB resection compared to wild-type cells (Fig 4D). Importantly, the lack of *TEL1*, which caused per se a slight delay in DSB resection (Fig 4D) [38], did not reduce further the resection efficiency of *rad50-V1269M* cells, as *rad50-V1269M* and *tel1*Δ *rad50-V1269M* cells resected the DSB with similar kinetics (Fig 4D). These findings indicate that the severe DNA damage sensitivity of *tel1*Δ *rad50-V1269M* double mutant cells is not due to a resection defect. Further supporting this conclusion, *EXO1* overexpression, which suppresses the hypersensitivity to DNA damaging agents and the DSB resection defect of *mre11*Δ cells [38,49], did not suppress the hypersensitivity to DNA damaging agents of *tel1*Δ *rad50-V1269M* double mutant cells (Fig 4E).

### The Lack of Tel1 Impairs MR^V1269M^X Function in End-Tethering

MRX function in end-tethering is largely dependent on Rad50 coiled-coil domains [12,14]. Nonetheless, structural studies suggest that also DNA binding of the globular domain is important for end-tethering, possibly because it increases intercomplex hook-hook dimer formation by causing the Rad50 coils to become more rigid and parallel to one another [12]. As the MR^V1269M^X mutant complex was poorly recruited to the DSB (Fig 3A and 3B), we asked whether *rad50-V1269M* and *tel1*Δ *rad50-V1269M* cells were defective in keeping the DSB ends tethered to each other. To detect the association of broken DNA ends, we used a yeast strain where the DNA proximal to an irreparable HO-induced DSB can be visualized by binding of a LacI-GFP fusion protein to multiple repeats of the LacI repressor binding site (LacO) that are integrated on both sides of the HO cleavage site on chromosome VII at a distance of 50 kb [43]. HO was induced by galactose addition to cell cultures that were arrested in G2 with nocodazole and kept blocked in G2 by nocodazole treatment in order to ensure that all cells would arrest in metaphase. The majority of wild-type cells showed a single LacI-GFP focus both before and after HO induction, indicating their ability to hold the broken DNA ends together (Fig 4F). Consistent with previous results [45], *tel1*Δ cells showed a slight increase of two LacI-GFP spots at 1–3 h after HO induction (Fig 4F). An increase of two LacI-GFP spots compared to the uninduced condition could be detected also in *rad50-V1269M* cells (Fig 4F). Strikingly, the number of cells showing two LacI-GFP spots after HO induction was greatly increased in *tel1*Δ *rad50-V1269M* double mutant cells compared to each single mutant, reaching a percentage similar to that observed in *mre11*Δ cells (Fig 4F).

The MRX complex has been implicated in sister chromatid cohesion [50], prompting us to evaluate whether the increase frequency of two LacI-GFP foci after HO induction in *tel1*Δ *rad50-V1269M* cells was due to end-tethering and/or cohesion defects. We therefore induced HO expression in α-factor-arrested cells that were kept arrested in G1 by α-factor in the presence of galactose. About 50% of G1-arrested *tel1*Δ *rad50-V1269M* cells showed two LacI-GFP foci 1 h after HO induction similarly to *mre11*Δ cells (Fig 4G), indicating that the appearance of two LacI-GFP foci in these cells is primarily due to defective end-tethering.

We also monitored the ability of *tel1*Δ, *rad50-V1269M*, and *tel1*Δ *rad50-V1269M* cells to maintain cohesion between sister chromatids by determining formation of LacI-GFP foci in nocodazole-arrested cells in the absence of HO induction. Under these conditions, the amount of *tel1*Δ and *rad50-V1269M* cells showing two LacI-GFP foci was similar to that found in wild-type cells (Fig 4H), indicating that cohesion is not affected by either the lack of Tel1 or the presence of Rad50-V129M variant. By contrast, a slight cohesion defect was detectable in nocodazole-arrested *tel1*Δ *rad50-V1269M* cells, which showed a ~5% increase of two LacI-GFP foci compared to wild-type cells (Fig 4H).

Because the ability of the above strains to held together the DSB ends was determined by using target sequences integrated at a distance of 50 kb from the DSB, we also monitored end-tethering by using a strain expressing LacI-YFP and TetR-RFP fusion proteins, which bind LacO and TetO tandem arrays, respectively [51]. These arrays are integrated at a distance of 7 kb from the DSB that is generated by the endonuclease HO on chromosome III, with each kind of array marking one specific side of the break. The frequency of cells showing separated LacI-YFP and TetR-RFP foci dramatically increased after HO induction in G2-arrested *tel1*Δ *rad50-V1269M* cells compared to wild-type cells (Fig 4I), confirming that *tel1*Δ *rad50-V1269M* cells are defective in end-tethering. Thus, the absence of Tel1 severely reduces the end-tethering activity of MR^V1269M^X, indicating a role for Tel1 in supporting this MRX function.

### The Lack of Tel1 Impairs MR^V1269M^X Function in DSB Repair by HR

The maintenance of the DSB ends tethered to each other is a relevant event in the repair of a DSB by both NHEJ and HR [43,44,46,52]. Thus, we asked whether *tel1*Δ *rad50-V1269M* cells were defective in HR and/or NHEJ. Among the HR pathways, single-strand annealing (SSA) is devoted to repair a DSB that is flanked by direct repeats and requires resection of the DSB ends followed by Rad52-dependent annealing of the resulting complementary ssDNA sequences [53]. To investigate possible HR defects, we first monitored the ability of *tel1*Δ *rad50-V1269M* cells to repair a DSB by SSA. To this end, we used a strain carrying a galactose-inducible *GAL-HO* construct, as well as tandem repeats of the *LEU2* gene, with a recognition site for the HO endonuclease adjacent to one of the repeats (S1A Fig) [54]. Galactose was added to G2-arrested cells to induce HO production and it was maintained in the medium so that continuously produced HO could re-cleave the HO sites eventually reconstituted by NHEJ. When kinetics of DSB repair was monitored by Southern blot analysis with a *LEU2* probe, accumulation of the 8 kb SSA repair product was slightly delayed in both *tel1*Δ and *rad50-V1269M* single mutants, whereas it was severely defective in *tel1*Δ *rad50-V1269M* double mutant compared to wild-type cells (S1B and S1C Fig). This finding indicates that Tel1 is important to support MRX function in DSB repair by SSA. The observation that *tel1*Δ, *rad50-V1269M* and *tel1*Δ *rad50-V1269M* cells all delay resection to the same extent (Fig 4D) indicates that the SSA defect of *tel1*Δ *rad50-V1269M* cells cannot be explained by a resection defect. We noticed that all the galactose-induced cell cultures exhibited a DNA band that migrated slower than the uncut band and appeared concomitantly with the SSA products (S1C Fig). This band was not detectable in *rad51*Δ cells, indicating that it was generated by Rad51-mediated recombination events (S1D Fig).

Because SSA repair pathway does not involve strand invasion and therefore does not require the recombination protein Rad51 (S1D Fig) [55], we also monitored the HR events that depend on the Rad51-dependent invasion and pairing of broken DNA ends with intact homologous sequences present on a sister chromatid or at an ectopic location in the genome. In the major HR pathway, the 3′-ended ssDNA tail invades an intact duplex homologous, creating a loop structure (D-loop) consisting of a region of heteroduplex DNA and displaced ssDNA. If this ssDNA anneals with the complementary sequence on the other side of the DSB (second end capture), subsequent extension and ligation result in the formation of a double Holliday junction intermediate, whose random cleavage yield an equal number of noncrossover (NCO) and crossover (CO) products [53]. Alternatively, if the newly synthesized strand is displaced, it can anneal with the 3′ ssDNA end at the other end of the DSB. This event leads to the generation of NCO products in a process called synthesis-dependent strand-annealing (SDSA) [56–58].

**Fig 5:**
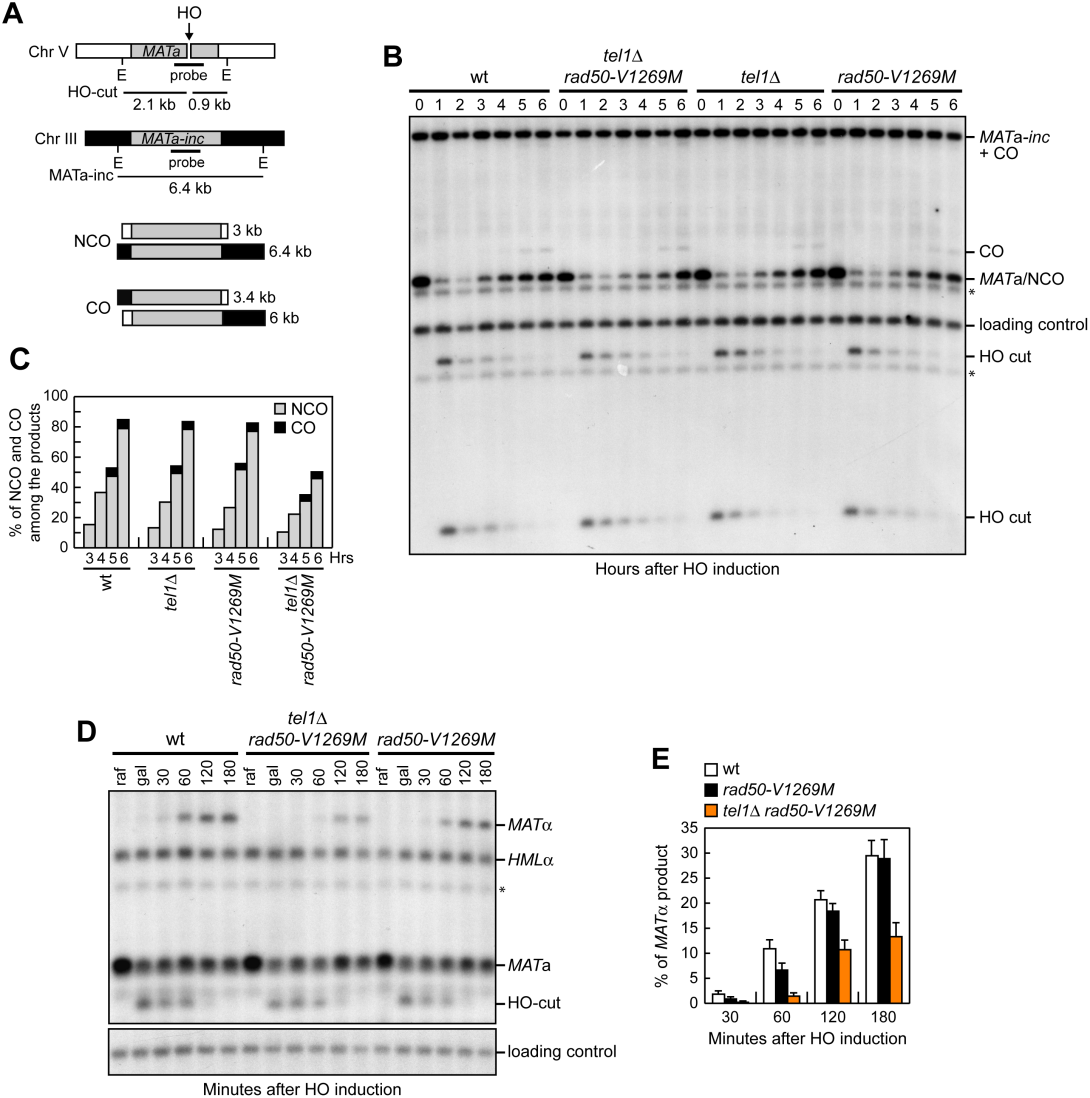
*rad50-V1269M tel1*Δ mutant cells are defective in DSB repair by HR. (A) System to detect ectopic recombination. HO generates a DSB at a *MAT*a DNA sequence inserted on chromosome V, while the homologous *MAT*a-*inc* region on chromosome III cannot be cut by HO and is used as a donor for HR-mediated repair, which can generate both noncrossover (NCO) and crossover (CO) products. E, EcoRI. (B) Exponentially growing YEPR cell cultures were transferred to YEPRG at time zero. Southern blot analysis of EcoRI-digested genomic DNA with the *MAT*a probe depicted in A. (C) Densitometric analysis of CO versus NCO repair bands at the indicated times after HO induction (see Materials and Methods). (D) Mating type switching. Exponentially growing YEPR *MAT*a cell cultures (raf) were transferred to YEPRG to induce HO. After 30 min (gal), cells were transferred to YEPD to allow mating type switching. StyI-BamHI-digested genomic DNA prepared at the indicated times after glucose addition was subjected to Southern blot analysis with a *MAT*a probe. (E) Densitometric analysis of the *MAT*α product band signals (see Materials and Methods). Plotted values are the mean value with error bars denoting s.d. (*n* = 3). * indicates cross hybridization signals.

To monitor CO and NCO formation, we used haploid strains that bear two copies of the *MAT*a sequence. A *MAT*a gene introduced in chromosome V can be cleaved by a galactose-inducible HO endonuclease and repaired by Rad51-dependent HR using a uncleavable *MAT*a donor on chromosome III that contains a single base pair substitution preventing HO cleavage (*MAT*a*-inc*) (Fig 5A) [59]. This repair event can lead to NCO and CO outcomes, with the proportion of COs being ~5% among the overall repair events [59]. Galactose was added to induce HO production and then it was maintained in the medium to cleave the HO sites that were eventually reconstituted by NHEJ-mediated DSB repair. The 3 kb *MAT*a band resulting from NCO recombination events re-accumulated less efficiently in *tel1*Δ *rad50-V1269M* double mutant cells compared to both *tel1*Δ and *rad50-V1269M* single mutant cells, which generated NCO products similar to wild-type cells (Fig 5B and 5C). Interestingly, while *tel1*Δ *rad50-V1269M* double mutant cells showed decreased amount of NCOs compared to wild-type cells, the percentage of COs in the same cells was similar to that observed in wild-type cells (Fig 5B and 5C). As most of the NCO products are generated by the SDSA mechanism, this finding suggests that *tel1*Δ *rad50-V1269M* cells are specifically defective in SDSA.

Because SDSA is thought to be the main mechanism responsible for mating type switching [60], we investigated the ability of *tel1*Δ *rad50-V1269M* cells to switch the mating type. HO expression was induced for 30 min by galactose addition to *MAT*a cells and was then rapidly shut off by the addition of glucose to allow repair of the HO-induced break by gene conversion. Since there is a strong mating type-dependent preference for the choice of the two silent donor loci *HML*α and *HMR*a [60], the *MAT*a sequence will be replaced preferentially with the *HML*α donor sequence to generate the *MAT*α product. Strikingly, *tel1*Δ *rad50-V1269M* cells accumulated the *MAT*α repair product less efficiently than *rad50-V1269M* cells, which generated this product with almost wild-type kinetics (Fig 5D and 5E). These findings indicate that *tel1*Δ *rad50-V1269M* double mutant cells are defective in mating type switching, supporting the hypothesis that they are specifically impaired in SDSA-based recombination mechanisms.

### The Lack of Tel1 Impairs MR^V1269M^X Function in DSB Repair by NHEJ

**Fig 6:**
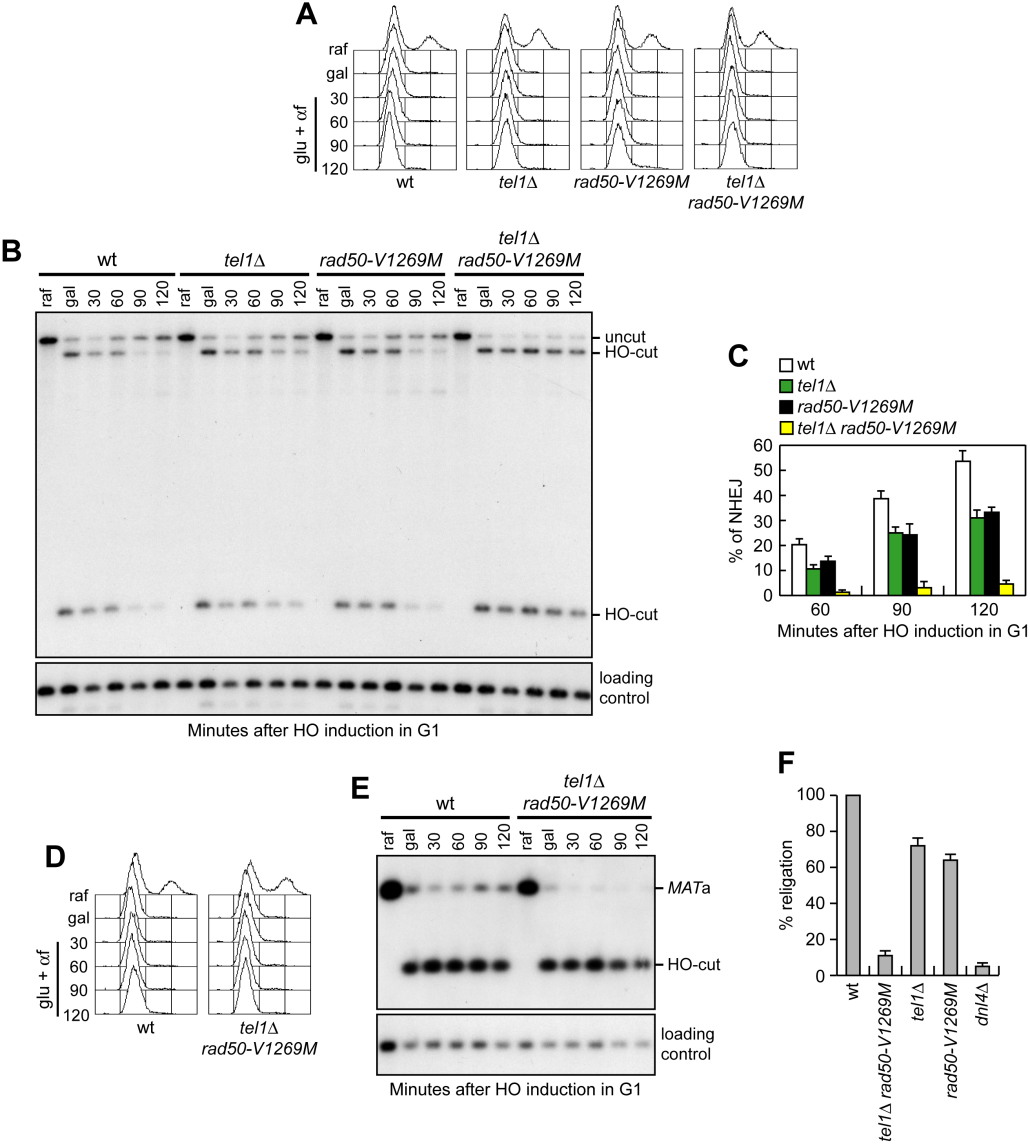
*tel1*Δ *rad50-V1269M* mutant cells are defective in DSB repair by NHEJ. (A, B) Exponentially growing YEPR cultures (raf) of cells carrying the system described in S1 Fig were arrested in G1 with α-factor and transferred to YEPRG in the presence of α-factor. After 30 min (gal), cells were transferred to YEPD medium in the presence of α-factor (glu+αf) to allow NHEJ. (A) FACS analysis of DNA content. (B) Southern blot analysis with a *LEU2* probe of KpnI-digested genomic DNA prepared at the indicated times after transfer to glucose. (C) Densitometric analysis of the uncut band signals (see Materials and Methods). Plotted values are the mean value with error bars denoting s.d. (*n* = 3). (D, E) Exponentially growing YEPR cultures (raf) of cells expressing a galactose-inducible HO endonuclease and lacking the homologous donor loci *HML*α and *HMR*a were arrested in G1 with α-factor and transferred to YEPRG in the presence of α-factor. After 30 min (gal), cells were transferred to YEPD medium in the presence of α-factor (glu+αf) to allow NHEJ. (D) FACS analysis of DNA content. (E) SspI-digested genomic DNA prepared at the indicated time points after glucose addition were subjected to Southern blot analysis with a *MAT*a probe. (F) Plasmid re-ligation assay. The same amounts of BamHI-linearized or uncut pRS316 plasmid DNA were transformed into the cells with the indicated genotypes, followed by transformant selection for the pRS316 nutritional marker. Data are expressed as percentage of re-ligation relative to wild type that was set up at 100% after normalization to the corresponding transformation efficiency of the uncut plasmid.

Next, we investigated whether *tel1*Δ *rad50-V1269M* cells were defective in NHEJ. To this purpose, we used the strains previously used to monitor DSB repair by SSA. HO expression was induced for 30 min by galactose addition and was then rapidly shut off by the addition of glucose to allow NHEJ-mediated repair of the DSB. To ensure that repair of the HO-induced DSB occurred mainly by NHEJ, HO was induced in G1-arrested cells that were kept arrested in G1 with α-factor (Fig 6A). In fact, the low Cdk1 activity in G1 cells prevents resection of the HO-induced DSB and therefore its repair by SSA [61,62]. NHEJ-mediated DSB repair was severely affected in *tel1*Δ *rad50-V1269M* cells. In fact, the 14.5 kb uncut band resulting from NHEJ-mediated ligation of the DSB ends failed to re-accumulate in *tel1*Δ *rad50-V1269M* cells compared to wild type (Fig 6B and 6C), which also showed the expected decrease of both the 2.5 and 12 kb HO-cut band signals due to DSB repair by NHEJ (Fig 6B). Some defective re-accumulation of the 14.5 kb uncut band could be detected also in both *rad50-V1269M* and *tel1*Δ single mutant cells, although this defect was much less severe compared to that observed in *tel1*Δ *rad50-V1269M* cells (Fig 6B and 6C).

To confirm the NHEJ defect, we used the *GAL-HO* strain, where HO induction by galactose addition generates a DSB at the *MAT*a locus. This strain lacks the homologous donor sequences *HML*α and *HMR*a and therefore can repair the HO-induced DSB only by NHEJ. HO expression was induced for 30 min by galactose addition to G1-arrested cells and then shut off by glucose addition to allow DSB repair by NHEJ (Fig 6D). The *MAT*a sequence resulting from NHEJ repair events re-accumulated in wild-type cells but not in *tel1*Δ *rad50-V1269M* double mutant cells (Fig 6E), thus confirming a severe NHEJ defect.

Finally, we measured NHEJ efficiency also as the ability of the above cells to re-ligate a plasmid that was linearized before being transformed into the cells [63]. Both *rad50-V1269M* and *tel1*Δ mutants showed only a slight reduction in the efficiency of plasmid re-ligation compared to wild-type cells (Fig 6F). By contrast, the re-ligation efficiency in *tel1*Δ *rad50-V1269M* cells dramatically decreased to a level similar to that found in *dnl4*Δ cells that lack the NHEJ enzyme responsible for DSB end ligation (Fig 6F). Thus, DSB repair by NHEJ, which is only slightly affected by the lack of Tel1 or the presence of the Rad50-V1269M mutant variant, is lost in *tel1*Δ *rad50-V1269M* double mutant cells, indicating that Tel1 supports MR^V1269M^X function also in NHEJ.

### Tel1 Promotes MRX Association to DNA Ends

**Fig 7:**
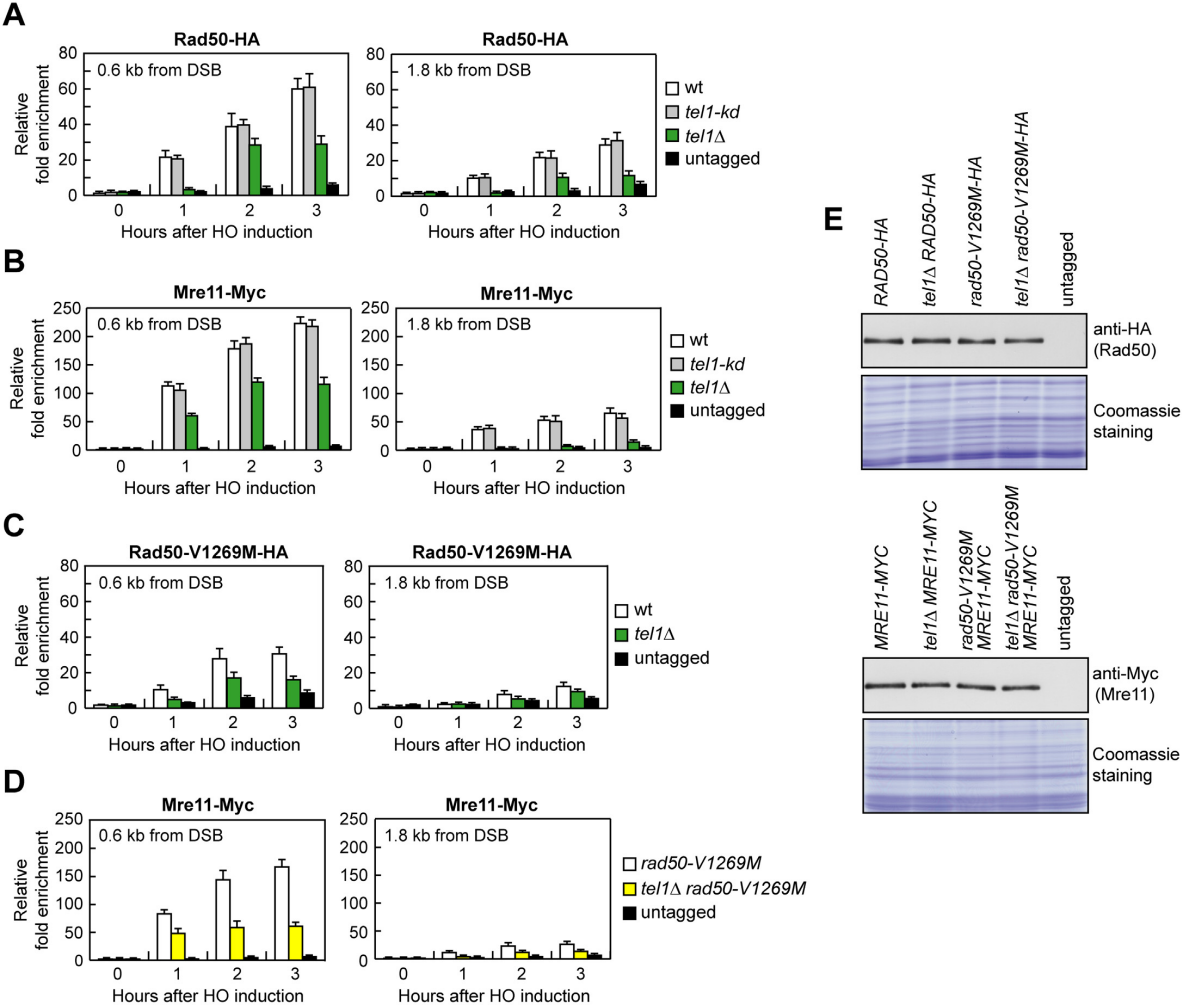
The lack of Tel1 impairs MRX and MR^V1269M^X association to a DSB. (A–D) ChIP analysis. Exponentially growing YEPR cell cultures were transferred to YEPRG at time zero. Relative fold enrichment of the indicated fusion proteins at the indicated distances from the HO cleavage site was determined after ChIP with anti-HA or anti-Myc antibodies and subsequent qPCR analysis. Plotted values are the mean values with error bars denoting s.d. (*n* = 3). (E) Western blot with anti-HA or anti-Myc antibodies of extracts used for the ChIP analysis shown in (A–D). The same amount of protein extracts was separated on a SDS-PAGE and stained with Coomassie Blue (loading control).

A structural study has proposed that DNA tethering requires proper binding to DNA of the MRX globular domain to induce intercomplex hook-hook dimer formation [12]. The finding that the lack of Tel1 exacerbates the end-tethering defects of *rad50-V1269M* cells raises the possibility that Tel1 supports MRX function in DNA tethering by promoting proper MRX association to damaged DNA. It has been previously shown that the lack of Tel1 decreases Mre11 binding at DNA ends flanked by telomeric DNA repeats [35,36], whereas only a slight reduction of Mre11 association, if any, can be detected in *tel1*Δ cells at 1 kb from an HO-induced DSB [35]. However, since only a limited amount of MRX is bound at this distance from the DSB (Fig 3A and 3B) [35], we analyzed Rad50 and Mre11 association at 0.6 kb from the DSB, where Mre11 is strongly enriched (Fig 3B). The amount of Rad50 (Fig 7A) and Mre11 (Fig 7B) bound at the HO-induced DSB ends was lower in *tel1*Δ cells than in wild-type cells, indicating that Tel1 promotes MRX association to DSBs. Consistent with the finding that the lack of Tel1 kinase activity did not exacerbate the DNA damage sensitivity of *rad50-V1269M* cells (Fig 1C), the association of Rad50 and Mre11 to DSBs was not affected in *tel1-kd* cells (Fig 7A and 7B). The lack of Tel1 also reduced the association to the DSB of Rad50-V1269M (Fig 7C) and Mre11 in *rad50-V1269M* cells (Fig 7D). The decreased DSB association of Rad50, Rad50-V1269M and Mre11 in *tel1*Δ compared to wild type was not due to reduced protein levels, as similar amounts of Rad50 and Mre11 could be detected in protein extracts prepared from wild-type, *tel1*Δ, *rad50-V1269M* and *tel1*Δ *rad50-V1269M* cells (Fig 7E). As MRX is required to load Tel1 on the DSB ends, these findings indicate that Tel1, once loaded onto the DSB by MRX, promotes MRX association/persistence in a feedback loop and this function is necessary to support the end-tethering activity of MRX.

### Rif2 Is Recruited to DSBs and Counteracts Tel1 Function in Promoting MRX Association to DSBs

**Fig 8:**
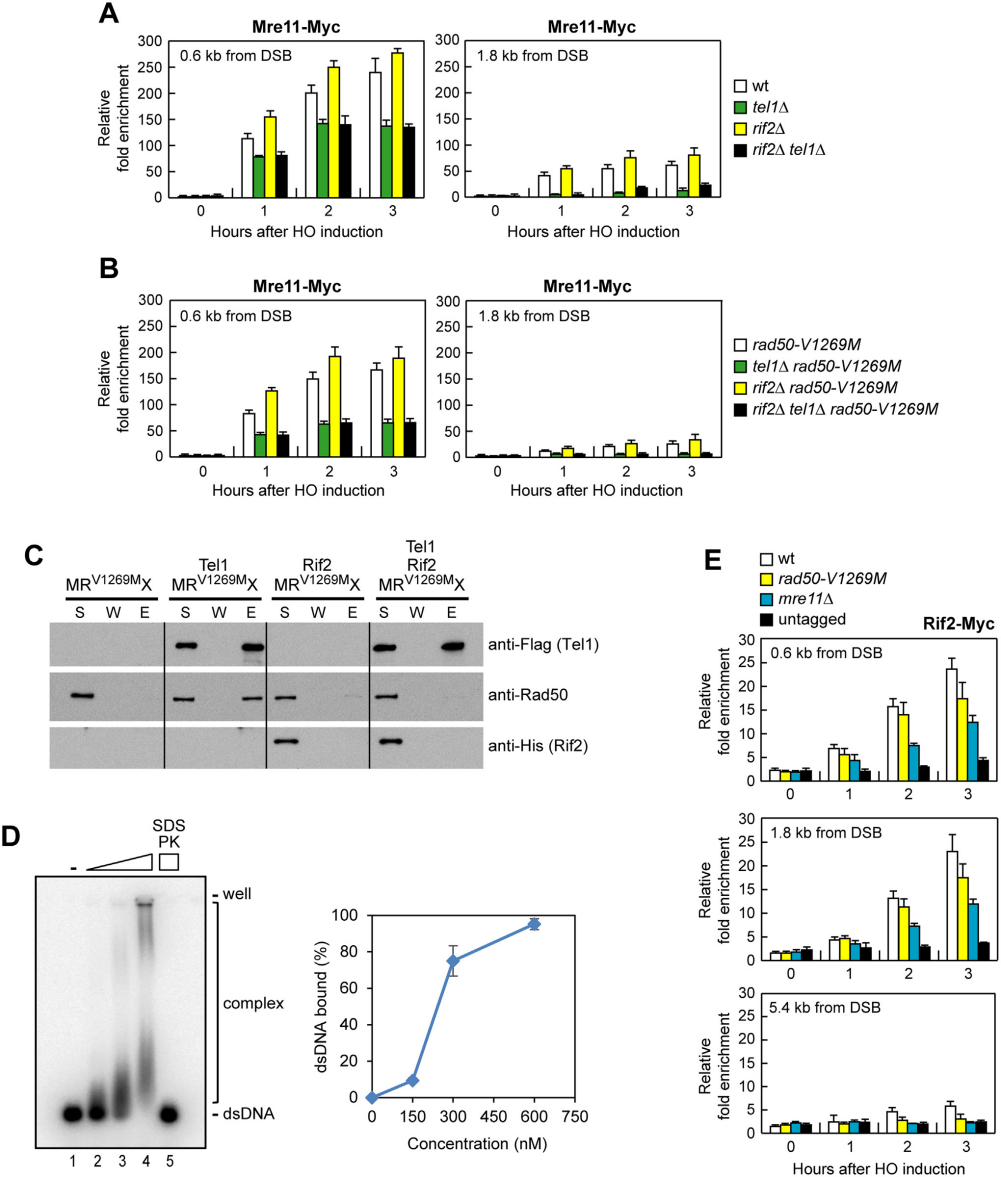
Rif2 is recruited to DNA ends, and its lack enhances MRX and MR^V1269M^X association to the DSB. (A, B) ChIP analysis. Exponentially growing YEPR cell cultures were transferred to YEPRG at time zero. Relative fold enrichment of Mre11-Myc fusion protein at the indicated distances from the HO cleavage site was determined after ChIP with anti-Myc antibodies and subsequent qPCR analysis. Plotted values are the mean values with error bars denoting s.d. (*n* = 3). (C) Flag-tagged Tel1 (50 ng) was incubated with MR^V1269M^X (100 ng) in the absence or presence of Rif2 (100 ng), and protein complexes were captured by anti-Flag resin and followed by immunoblotting analysis. (D) Rif2 (150, 300 and 600 nM) was incubated with ^32^P-labeled 100-bp dsDNA (10 nM) in the presence of ATP. In lane 5, the reaction mixture was deproteinized with SDS and proteinase K (PK) prior to analysis. Plotted values are the mean value with error bars denoting s.d. (*n* = 3). (E) ChIP analysis. As in (A), but showing Rif2 recruitment at the HO-induced DSB.

Rif2 was shown to counteract MRX association at telomeres by inhibiting the recruitment of Tel1, which in turn promotes MRX accumulation at telomere ends [35]. We asked whether Rif2 can modulate MRX association also at intrachromosomal DSBs by investigating the effect of *RIF2* deletion on MRX binding at the HO-induced DSB in *tel1*Δ, *rad50-V1269M*, and *tel1*Δ *rad50-V1269M* cells. ChIP and qPCR analysis showed that the amount of MRX (Fig 8A) and MR^V1269M^X (Fig 8B) bound at the HO-induced DSB ends was slightly higher in the absence than in the presence of Rif2, although similar amount of Mre11 could be detected in protein extracts prepared from wild-type, *rif2*Δ, and *rif2*Δ *rad50-V1269M* cells (S2 Fig). This finding indicates that Rif2 counteracts MRX and MR^V1269M^X association to DSBs.

This Rif2 function was completely dependent on Tel1, as the amount of both MRX (Fig 8A) and MR^V1269M^X (Fig 8B) bound at the DSB decreased to similar levels in both *tel1*Δ and *tel1*Δ *rif2*Δ cells. Consistent with a previous finding that Rif2 competes with Tel1 for the binding to MRX [35], the interaction between Tel1 and MR^V1269M^X was strongly attenuated by Rif2 (Fig 8C). All together, these data suggest that Rif2 inhibits MRX association at DSBs by counteracting MRX-Tel1 interaction.

Consistent with a direct role of Rif2 in DSB metabolism, purified Rif2 turned out to bind dsDNA in vitro (Fig 8D). Furthermore, following HO induction by galactose addition, a fully functional Myc-tagged Rif2 variant was efficiently recruited close to the HO-induced DSB and its binding increased over 3 h, spreading to 2 kb from the HO cleavage site (Fig 8E). As Rif2 physically interacts with MRX [35], we asked whether MRX may contribute to Rif2 binding at the DSB. Indeed, the amount of Rif2 bound at the DSB decreased in both *rad50-V1269M* and *mre11*Δ cells (Fig 8E), indicating that Rif2 association to the DSB is partially dependent on MRX.

### The Lack of Rif2 Restores MR^V1269M^X Function in End-Tethering

**Fig 9:**
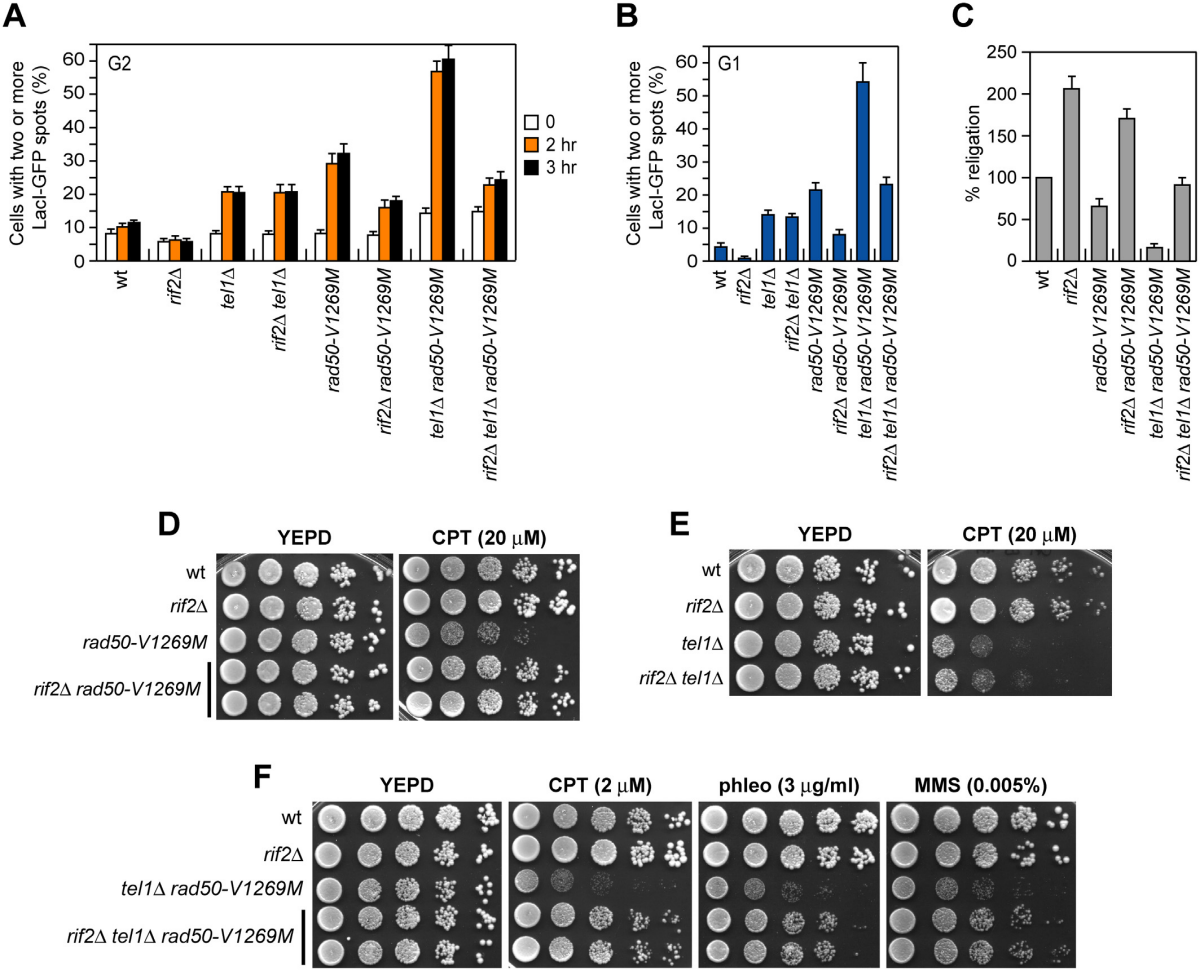
Rif2 attenuates MRX function in end-tethering and NHEJ. (A, B) DSB end-tethering. The assay was performed as described in Fig 4F and 4G. Plotted values are the mean value with error bars denoting s.d. (*n* = 3). (C) Plasmid re-ligation assay. The assay was done as described in Fig 6F. (D–F) Exponentially growing cultures were serially diluted (1:10) and each dilution was spotted out onto YEPD plates with or without CPT, phleomycin, or MMS at the indicated concentrations.

The finding that the lack of Rif2 increases MRX and MR^V1269M^X association to DSBs prompted us to ask whether *RIF2* deletion could restore end-tethering in *rad50-V1269M* mutant cells. Indeed, *RIF2* deletion suppressed the end-tethering defects of G1- and G2-arrested *rad50-V1269M* cells (Fig 9A and 9B) and this suppression is specific for *rad50-V1269M*, as *rif2*Δ did not restore end-tethering in G1- and G2-arrested *tel1*Δ cells (Fig 9A and 9B). As a consequence, the lack of Rif2 also rescued the NHEJ defects of *rad50-V1269M* cells, as *rif2*Δ *rad50-V1269M* cells re-ligated the BamHI-cut plasmid more efficiently than *rad50-V1269M* cells (Fig 9C).

Interestingly, both *rad50-V1269M* and *tel1*Δ single mutant cells did not lose viability in the presence of phleomycin, MMS or low CPT doses (Fig 1B), but they exhibited hypersensitivity to high doses of CPT (Fig 9D and 9E). Consistent with a role of Rif2 in limiting MRX functions, *rif2*Δ also suppressed the CPT hypersensitivity of *rad50-V1269M* (Fig 9D), but not that of *tel1*Δ mutant cells (Fig 9E).

The lack of Rif2 increased the association of MRX and MR^V1269M^X at DSBs in a Tel1-dependent manner (Fig 8A and 8B), indicating that Rif2 counteracts the ability of Tel1 to enhance MRX association to DSB. If the lack of Rif2 suppressed the CPT hypersensitivity and the end-tethering defect of *rad50-V1269M* cells by increasing the amount of MR^V1269M^X bound to the DSB, this *rif2*Δ-mediated suppression should require Tel1 and therefore *rif2*Δ should not be able to suppress the same defects in *tel1*Δ *rad50-V1269M* cells. However, we found that *rif2*Δ restored both end-tethering (Fig 9A and 9B) and NHEJ (Fig 9C), as well as DNA damage resistance (Fig 9F) also of *tel1*Δ *rad50-V1269M* double mutant cells. This finding indicates that the restored DNA damage resistance and end-tethering in *tel1*Δ *rif2*Δ *rad50-V1269M* cells do not simply depend on increased amount of MRX bound at the DSB.

### Rif2 Enhances MRX ATPase Activity and Limits End-Tethering

**Fig 10:**
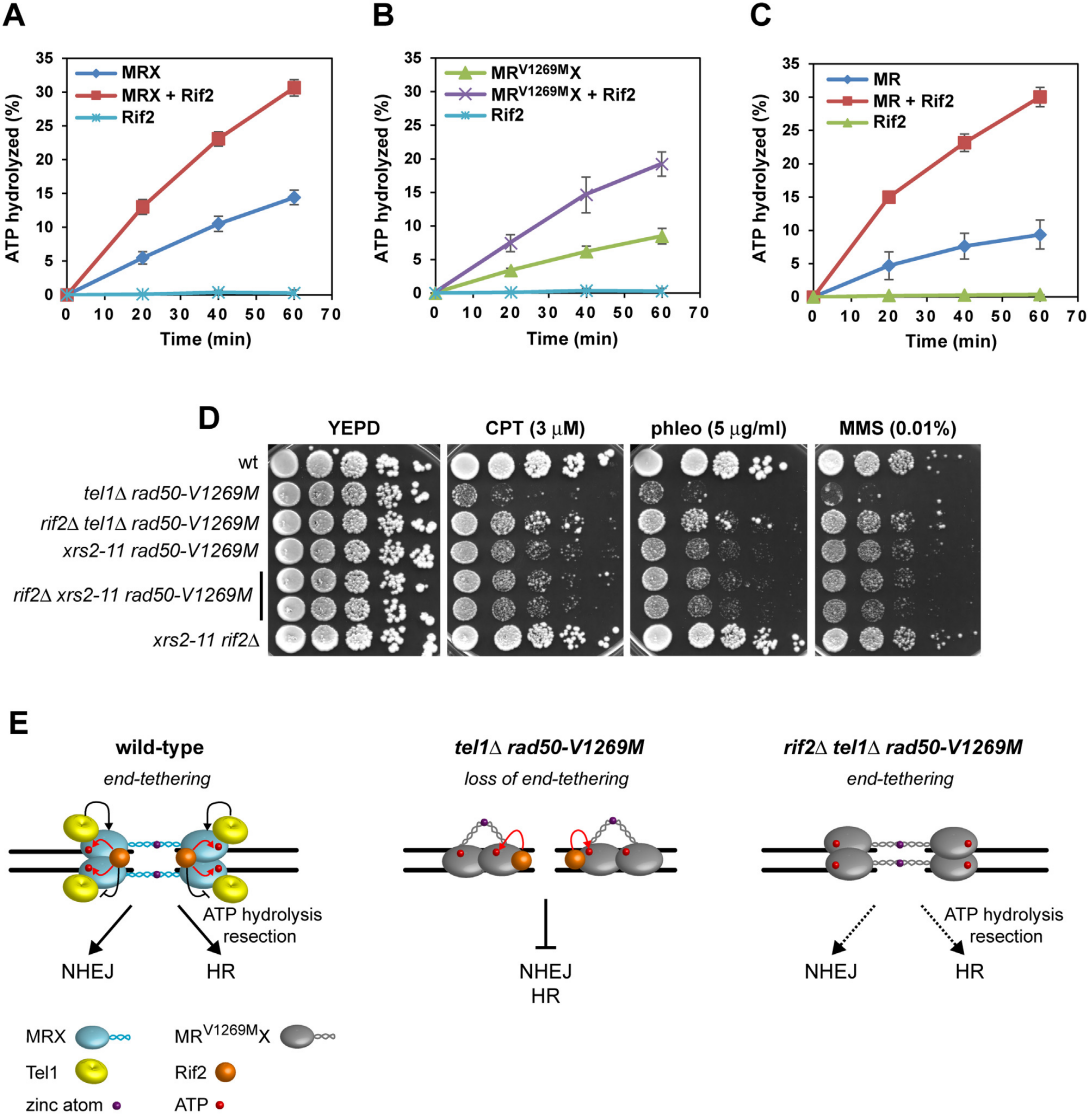
Rif2 enhances MRX ATPase activity. (A–C) The ATPase activity of wild-type MRX, MR^V1269M^X and MR complexes was determined in the absence or presence of Rif2. Plotted values are the mean value with error bars denoting s.d. (*n* = 3). (D) Exponentially growing cultures were serially diluted (1:10) and each dilution was spotted out onto YEPD plates with or without CPT, phleomycin, or MMS. (E) Model for the roles of Tel1 and Rif2 in controlling MRX function at DSBs. In wild-type cells (left), Tel1 enhances MRX function by promoting MRX association to DSBs (black arrows). Proper MRX accumulation to DNA increases the number of productive intercomplex interactions between hook domains to maintain DNA strands in close proximity. Rif2 inhibits MRX accumulation at DSBs by counteracting MRX-Tel1 interaction (black bar-headed line) and enhances MRX ATPase activity (red arrows). In *tel1*Δ *rad50-V1269M* cells (middle), the reduced amount of MR^V1269M^X bound at the DSB severely impairs end-tethering, HR and NHEJ. In *rif2*Δ *tel1*Δ *rad50-V1269M* cells (right), the lack of Rif2 restores end-tethering, HR and NHEJ by increasing the time spent by MRX in the ATP-bound state. The MR^V1269M^X complex is indicated in grey.

The finding that the lack of Rif2 restores DNA damage resistance and end-tethering in *rad50-V1269M* cells even in the absence of Tel1 suggests that Rif2 has other functions in regulating MRX activity besides limiting MRX recruitment to DNA ends. It has been proposed that ATP hydrolysis induces the change from a closed MRX complex, required for end-tethering, to an open configuration that promotes Mre11 nuclease activity and DSB resection [29]. Thus, we asked whether Rif2 attenuates MRX function in end-tethering by influencing its ATPase activity. The ATPase assay was performed in the presence of 200 nM of 100-bp double-stranded DNA (dsDNA) to fully activate MRX ATPase activity. Since efficiently shifting 10 nM of the same DNA requires at least 300 nM Rif2 (Fig 8D), 2 μM Rif2 was used to investigate the potential effect of Rif2-mediated DNA binding on ATP hydrolysis by MRX in reactions that contained 200 nM DNA. We found that the addition of purified Rif2 increased the ATP hydrolysis activity by both wild-type MRX (Fig 10A) and MR^V1269M^X complexes (Fig 10B). Since end-tethering requires the MRX ATP-bound state [29], this finding suggests that the lack of Rif2 suppresses the hypersensitivity to DNA damaging agents and the end-tethering defect of *rad50-V1269M* and *rad50-V1269M tel1*Δ cells by increasing the time spent by MRX in an ATP-bound closed conformation.

As an earlier study has shown that Rif2 binds to the C-terminus of Xrs2 [35], we asked whether this Rif2-mediated regulation of MRX activity requires Rif2-MRX interaction. Interestingly, the lack of Rif2 cannot suppress the hypersensitivity to DNA damaging agents of *rad50-V1269M* cells carrying the *xrs2-11* mutation (Fig 10D), which causes the lack of the Xrs2 C-terminal part and therefore of the MRX-Rif2 interaction. This finding suggests that regulation of MRX function by Rif2 requires its interaction with Xrs2. This requirement can be bypassed in vitro where these proteins are already in close proximity, as Rif2 can also enhance the ATPase activity of the MR complex (Fig 10C).

Mutants that promote the ATP-bound closed conformation of Rad50 exhibit a higher degree of tethering activity [29], and Rif2 enhances ATPase activity not only of MR^V1269M^X, but also of wild-type MRX (Fig 10A and 10B). If this Rif2 function is physiologically relevant also in a wild-type context, then *rif2*Δ cells should show improved efficiency of DNA tethering. We found that the percentage of *rif2*Δ cells showing two LacI-GFP spots was reproducibly decreased compared to wild-type cells, indicating that the tethering efficiency is higher in *rif2*Δ cells than in wild type (Fig 9A and 9B). Furthermore, Rif2 limits DSB repair by NHEJ, as *rif2*Δ mutant cells re-ligated the BamHI-cut plasmid more efficiently than wild-type cells (Fig 9C). These observations, together with the finding that Rif2 enhances ATP hydrolysis by MRX, suggest that Rif2 regulates MRX function by promoting ATP-driven Rad50 conformational changes.

## Discussion

We provide evidence that Tel1, once loaded to a DSB by the MRX complex, promotes/stabilizes MRX association to the DSB in a positive feedback loop. Tel1 exerts this function independenly of its kinase activity, suggesting that it plays a structural role in promoting/stabilizing MRX retention to DSBs. This Tel1-mediated control of MRX association can be important to ensure that MRX binding to DNA is end-specific and becomes crucial for cell viability after genotoxic treatment when MRX accumulation at DSBs is suboptimal, such as in *rad50-V1269M* mutant cells. The *rad50-V1269M* mutation impairs MRX association at DSBs and the lack of Tel1 reduces further the amount of MR^V1269M^X bound at DSBs. As a consequence, *tel1*Δ *rad50-V1269M* double mutant cells are much more sensitive to genotoxic agents compared to each single mutant. This DNA damage hypersensitivity is not due to defective DSB resection. Instead, *tel1*Δ *rad50-V1269M* cells are severely defective in keeping the DSB ends tethered to each other and in repairing a DSB by HR and NHEJ. Since the mainteinance of the DSB ends in close proximity is a relevant event in the repair of DSBs by both NHEJ and HR [43,44,46,52], the low degree of end-tethering in *tel1*Δ *rad50-V1269M* cells can explain the poor ability of the same cells to repair a DSB by both repair mechanisms.

During HR, the second DSB end can be captured by the D-loop to form an intermediate with double Holliday junctions, whose random cleavage results in equal number of NCO or CO products. However, if the newly synthesized strand is displaced by the D-loop and anneals to the other DSB end, this results in NCO products by the SDSA mechanism [53]. Consistent with the finding that only some MRX functions are lost in *tel1*Δ *rad50-V1269M* cells, COs are generated with wild-type kinetics in *tel1*Δ *rad50-V1269M* cells, whereas formation of these repair products are impaired by the lack of any MRX subunit [64]. Interestingly, *tel1*Δ *rad50-V1269M* cells are specifically defective in the generation of NCO products, suggesting that these cells can be specifically impaired in SDSA. This observation raises the possibility that the function of MRX in keeping the DSB ends in close proximity can be particularly important to facilitate the annealing of the displaced strand to the other DSB end. By contrast, this function can be escaped when the second DSB end is already captured by the D-loop and the DNA intermediate is stabilized by the formation of a double Holliday junction.

MRX association to DNA has been shown to induce parallel orientation of the Rad50 coiled-coils that favours intercomplex association needed for DNA tethering [12]. Our results support a model wherein Tel1, once loaded at DSBs by MRX, exerts positive feedback by promoting an end-specific association of MRX with DNA (Fig 10E). This Tel1-mediated regulation of DNA-MRX retention is important for proper MRX conformation needed for the tethering of broken DNA ends. Previous data have shown that the lack of Sae2 impairs end-tethering and increases MRX association to DSB ends [65,66]. These and our findings suggest that it is not the amount of MRX bound at DNA ends per se that simply dictates the integrity of end-tethering. Instead, a proper MRX-DNA interaction is required to allow the establishment of a productive MRX intercomplex association that is needed to maintain DNA strands in close proximity.

The amount of MRX and Tel1 bound at telomeres is lower than that found at DSBs [36]. This difference is due to a Rif2-mediated inhibition of Tel1 accumulation at telomeric ends, which has been proposed to protect telomeric DNA ends from over-elongation and checkpoint activation [35,36,67]. This Rif2 function in modulating MRX activity is not restricted to telomeric DNA ends. In fact, although the amount of Rif2 bound at an HO-induced DSB flanked by telomeric repeats is higher than that found at HO-induced DSB containing no telomeric sequences [35], we show that the lack of Rif2 increases the association of MRX in a Tel1-dependent manner also to intrachromosomal DNA ends. As Rif2 competes in vitro with Tel1 for the binding to MRX, Rif2 can limit MRX association to DSBs by reducing MRX-Tel1 interaction. Consistent with a direct role of Rif2 at DSBs, Rif2 can bind DNA both in vitro and in vivo and its binding at DSBs is partially dependent on MRX.

We also found that the lack of Rif2 suppresses the DNA damage sensitivity and the end-tethering defects of *tel1*Δ *rad50-V1269M* double mutant cells. As *rif2*Δ increases MRX association to DSBs only in the presence of Tel1, the finding that Tel1 is not required for *rif2*Δ*-*mediated suppression of the *rad50-V1269M* phenotypes suggests that Rif2 has other functions in regulating MRX activity besides limiting its association to DNA ends. Based on the characterization of Rad50 variants that either promote or destabilize the ATP-bound state, it has been proposed that the ATP-bound conformation of MRX promotes end-tethering, whereas release from this ATP-bound state by ATP hydrolysis is necessary to allow access to DNA of the Mre11 nuclease active site and subsequent DSB resection [29]. Our data show that Rif2 enhances the ATP hydrolysis activity of the MRX complex, suggesting that the lack of Rif2 might restore end-tethering and DNA damage resistance in *tel1*Δ *rad50-V1269M* cells by increasing the time spent by MRX in the ATP-bound closed conformation. Consistent with this hypothesis, *rif2*Δ cells show an increased efficiency of both end-tethering and NHEJ compared to wild-type cells. The finding that the lack of Rif2 suppresses the end-tethering defect and the DNA damage hypersensitivity of *tel1*Δ *rad50-V1269M* cells without increasing MRX association at DSBs suggests that the transition between closed and open MRX conformations does not necessarily result in different amounts of MRX bound at DSBs.

Thus, we propose that Rif2 has a dual function in regulating MRX functions at DSBs: (i) it counteracts MRX association at DSBs by inhibiting MRX-Tel1 interaction; (ii) it enhances MRX ATPase activity, promoting the transition of the complex from a closed state, required for tethering, to an open state that unmasks Mre11 nuclease active sites and thus is competent for DSB resection (Fig 10E). Interestingly, Rif2 is known to counteract NHEJ at telomeres [68]. Whether this Rif2 function depends on a Rif2-mediated regulation of MRX conformational changes is an interesting question that remains to be addressed.

Cancer therapies targeting ATM/Tel1 have been developed to increase the effectiveness of standard genotoxic treatments and/or to set up synthetic lethal approaches in cancers with DNA repair defects [69]. Interestingly, analysis of the mutational landscape in 7,494 sequenced tumors across 28 tumor types revealed that approximately 4% of all human tumors harbor mutations in the MRN/MRX complex [70]. Our finding that MRX dysfunctions can be rendered synthetically lethal with *tel1*Δ in the presence of genotoxic agents suggests that ATM inhibitors in combination with DNA-damaging chemotherapy could be beneficial in patients whose tumors are defective in MRN function.

## Materials and Methods

### Yeast Strains

Strain genotypes are listed in S1 Table. Strains JKM139 and YMV45 were kindly provided by J. Haber (Brandeis University, Waltham, United States). Strains JYK40.6 and W4441-11C, used to detect end-tethering, were kindly provided by D. P. Toczyski (University of California, San Francisco, US) and M. Lisby (University of Copenhagen, Denmark), respectively. Strain tGI354, used to detect ectopic recombination, was kindly provided by J. Haber. Cells were grown in YEP medium (1% yeast extract, 2% bactopeptone) supplemented with 2% glucose (YEPD), 2% raffinose (YEPR) or 2% raffinose and 3% galactose (YEPRG). Gene disruptions were generated by one-step PCR disruption method. All the synchronization experiments have been performed at 26°C.

### Search for Mutations That Sensitize *tel1*Δ Cells to DNA Damaging Agents

To search for mutations that sensitize *tel1*Δ cells to DNA damaging agents, *tel1*Δ cells were mutagenized with ethyl methanesulfonate and plated on YEPD plates. Approximately 100,000 survival colonies were replica plated on YEPD plates with or without phleomycin or CPT. Phleomycin and/or CPT sensitive clones were recovered and transformed with plasmid containing wild-type *TEL1* to identify those that lost DNA damage hypersensitivity. The corresponding original clones were then crossed to wild-type cells to identify by tetrad analysis the clones in which the increased sensitivity to DNA damaging agents was due to the simultaneous presence of *tel1*Δ and a single-gene mutation. Subsequent genetic analyses of the positive clones allowed grouping them in three allelic classes. In one class, the mutation responsible for the *tel1*Δ hypersensitivity to CPT and phleomycin was identified by genome sequencing and genetic analyses. To confirm that the *rad50-V1269M* mutation was responsible for the hypersensitivity to DNA damaging agents of *tel1*Δ cells, a *KANMX* gene was integrated downstream of the *rad50-V1269M* stop codon and the resulting strain was crossed to *tel1*Δ cells to verify by tetrad dissection that the increased sensitivity to CPT and phleomycin of *tel1*Δ cells co-segregated with the *KANMX* allele.

### DSB Resection

DSB end resection at the *MAT* locus in JKM139 derivative strains was analyzed on alkaline agarose gels, by using a single-stranded probe complementary to the unresected DSB strand, as previously described [71]. This probe was obtained by in vitro transcription using Promega Riboprobe System-T7 and plasmid pML514 as a template. Plasmid pML514 was constructed by inserting in the pGEM7Zf EcoRI site a 900-bp fragment containing part of the *MAT*α locus (coordinates 200870 to 201587 on chromosome III).

### DSB Repair by NHEJ and SSA

DSB repair by NHEJ and SSA in YMV45 strains were detected by Southern blot analysis using an *Asp*718-*Sal*I fragment containing part of the *LEU2* gene as a probe, as previously described [71,72]. To determine the efficiency of NHEJ, the intensity of the uncut band at 30 min after HO induction (maximum efficiency of DSB formation), normalized respect to a loading control, was subtracted to the normalized values of the same band at the subsequent time points after glucose addition. The obtained values were divided by the normalized intensity of the uncut band in raffinose (100%). To determine the efficiency of DSB repair by SSA, the normalized intensity of the SSA product band at different time points after HO induction was divided by the normalized intensity of the uncut band at time zero before HO induction (100%). The loading control was obtained by hybridization of the filters with a probe that anneals to the *RAD52* gene. DSB repair by NHEJ in JKM139 strains was detected by Southern blot analysis of SspI-digested genomic DNA with a *MAT*a probe (200870–201587 coordinates of chromosome III).

### Mating Type Switching

Strain W303 was transformed with a plasmid carrying HO under the control of a galactose inducible promoter. Mating type switching was detected by Southern blot analysis using a *MAT*a probe (201082–201588 coordinates of chromosome III). This probe is complementary also to the *HML*α locus (13826–13918 coordinates of chromosome III). To determine the efficiency of mating type switching, the intensity of the *MAT*α band at different time points after glucose addition, normalized respect to a loading control, was divided by the normalized intensity of the uncut *MAT*a band in raffinose (100%).

### DSB Repair by Ectopic Recombination

DSB repair by ectopic recombination was detected by using the tGI354 strain as described in [72]. To determine the repair efficiency, the intensity of the uncut band at 2 h after HO induction (maximum efficiency of DSB formation), normalized respect to a loading control, was subtracted to the normalized values of NCO and CO bands at the subsequent time points after galactose addition. The obtained values were divided by the normalized intensity of the uncut *MAT*a band at time zero before HO induction (100%).

### Plasmid Religation Assay

The centromeric pRS316 plasmid was digested with the BamHI restriction enzyme before being transformed into the cells. Parallel transformation with undigested pRS316 DNA was used to determine the transformation efficiency. Efficiency of re-ligation was determined by counting the number of colonies that were able to grow on medium selective for the plasmid marker and was normalized respect to the transformation efficiency for each sample. The re-ligation efficiency in mutant cells was compared to that of wild-type cells that was set up to 100%.

### ChIP Analysis

ChIP analysis was performed as previously described [73]. Input and immunoprecipitated DNA were purified and analyzed by qPCR using a Biorad MiniOpticon. Data are expressed as fold enrichment at the HO-induced DSB over that at the non-cleaved *ARO1* locus, after normalization of each ChIP signals to the corresponding input for each time point. Fold enrichment was then normalized to the efficiency of DSB induction.

### Protein Expression and Purification

All the protein purification steps were carried out at 0–4°C. Rad50, Mre11 and Xrs2 were overexpressed in yeast and purified as described previously [18,41]. The Rad50-V1269M mutant was expressed and purified with a similar yield using the procedure devised for the wild-type protein. To assemble the MRX complex [74], Rad50, Mre11, and Xrs2 were incubated together for 5 h on ice. The resulting MRX complex was separated from unassembled proteins in a Sephacryl S-400 gel filtration column.

Tel1 was overexpressed in the protease deficient yeast strain BJ5464 (*MAT*a *ura3-52 trp1 leu2*Δ*1 his3*Δ*200 pep4*::*HIS3 prb1*Δ*1*.*6R can1 GAL*) using pGAL-FLAG-Tel1 (a kind gift from K. Sugimoto). An overnight yeast culture was diluted 1:100 into 8 L of omission medium with 2% raffinose. Cells were further cultured at 30°C until the OD_660_ reached 0.8, when 2% galactose was added to induce Tel1 expression. Cells were cultured for another 16 h before harvest. The pellet (~40 g), after being agitated with dry ice in a coffee grinder, was resuspended in 40 ml of ice-cold lysis buffer (40 mM KH_2_PO_4_, pH 7.4, 20% glycerol, 1 mM EDTA, 0.1% NP-40, 2 mM DTT, 600 mM KCl, and a cocktail of protease inhibitors consisting of aprotinin, chymostatin, leupeptin, and pepstatin A at 5 μg/ml each, and also 1 mM phenyl-methylsulfonyl fluoride). The lysate was clarified by centrifugation (20,000 xg, 30 min) and the supernatant was incubated with 0.5 ml of anti-FLAG-M2 agarose resin for 2 h. After washing the matrix with 30 ml of K buffer (20 mM KH_2_PO_4_, pH 7.4, 10% glycerol, 0.5 mM EDTA, 0.01% NP-40, 1 mM DTT) with 500 mM KCl, Tel1 was eluted with 1 ml of K buffer with 500 mM KCl and 200 μg/ml FLAG peptide for 1 h. The eluate containing purified Tel1 was concentrated and filter dialyzed against K buffer with 500 mM KCl in an Ultracel-30K micro-concentrator (Amicon). Purified Tel1 was stored at -80°C in small aliquots.

To express (His)_6_-tagged Rif2, the pET-Rif2 plasmid (a kind gift from K. Sugimoto) was introduced into BL21 Rosetta cells. Early log phase culture was treated with 0.3 mM IPTG to induce Rif2 expression. After 4 h of incubation at 37°C, cells were harvested and Rif2 was purified using the following procedure. Briefly, the clarified cell lysate from 38 g pellet prepared by sonication in 100 ml T buffer (25 mM Tris-HCl, pH 7.5, 10% glycerol, 0.5 mM EDTA, 0.01% Igepal, and 1 mM DTT) containing 300 mM KCl and the protease inhibitor cocktail was mixed gently with 6 ml Ni-NTA resin (Qiagen) to capture the (His)_6_-tagged Rif2. After washing extensively with 100 ml T buffer containing 1M KCl and 20 mM imidazole, bound proteins were eluted with 8 ml T buffer containing 150 mM KCl and 200 mM imidazole. The eluate was applied onto a SP Sepharose column (6 ml), which was developed with a 90 ml gradient of 50–350 mM KCl in T buffer. The peak fractions containing (His)_6_-Rif2 were pooled and then applied onto a Mono S column (1 ml), which was developed with a 30 ml gradient of 75–450 mM KCl in T buffer. After concentrating the pooled peak fractions to 1.5 ml in an Ultracel-30K concentrator (Amicon), the preparation (0.5 ml protein) was further fractionated in a Superdex 200 column (24 ml) in T buffer with 300 mM KCl. The highly purified (His)_6_-Rif2 protein (1 mg) was concentrated and stored in small portions at -80°C.

### ATPase and DNA Binding Assays

The ATPase assay was performed as described previously with the level of ATP hydrolysis being measured by thin layer chromatography and phosphorimaging analysis [27]. Briefly, wild-type MRX or MR^V1269M^X (100 nM) and Rif2 (2 μM) were used in the presence of 100-bp dsDNA (200 nM). The effect of Rif2 on the ATPase activity of the MR complex (100 nM) was examined as described above.

The DNA binding assay for Rad50 and MRX was slightly modified from that published previously [27]. Briefly, wild-type Rad50 and Rad50-V1269M (50, 100, and 200 nM), wild-type MRX and MR^V1269M^X (10, 20, and 40 nM) were incubated with radiolabeled 70-bp dsDNA substrate (10 nM) in the reaction buffer (25 mM Tris-HCl, pH 7.5, 150 mM KCl, 2 mM MgCl_2_, 1 mM DTT, 100 μg/ml BSA, 2 mM ATP) at 30°C for 10 min. The reaction mixtures were resolved in a 0.3% agarose gel in SB buffer (10 mM NaOH, 40 mM boric acid, pH 8.0) on ice. The gel was dried and then subject to phosphorimaging analysis. To examine Rif2 for DNA binding activity, the indicated amount of purified Rif2 was incubated with radiolabeled 100-bp dsDNA substrate (10 nM) in the reaction buffer at 30°C for 10 min, followed by the same analytical procedure described above.

### Affinity Pull-Down Assay

Flag-tagged Tel1 was incubated with MRX or MR^V1269M^X in 30 μl T buffer containing 100 mM KCl for 2 h on ice. The reaction was mixed gently with anti-FLAG-M2 agarose resin (10 μl) for 2 h to capture Flag-tagged Tel1 and associated MRX or MR^V1269M^X. After washing the resin three times with 200 μl T buffer, bound proteins were eluted with SDS-PAGE loading buffer and then subject to western blot analysis. To determine the effect of Rif2 on the interaction between Tel1 and MR^V1269M^X, Flag-tagged Tel1 was incubated with MR^V1269M^X in the absence or presence of Rif2, and then subjected to affinity pull-down as described above.

## Supporting Information

S1 Data Numerical data for all the graphs. (XLSX)

S1 Fig DSB repair by SSA. (A) Map of the YMV45 chromosome III region where the HO-cut site is flanked by homologous *leu2* sequences (black boxes) that are 4.6 kb apart. HO-induced DSB formation results in generation of 12 kb and 2.5 kb DNA fragments (HO-cut) that can be detected by Southern blot analysis of KpnI-digested genomic DNA with a *LEU2* probe. DSB repair by SSA generates a product of 8 kb (SSA). K, KpnI. (B) DSB repair by SSA. YEPR exponentially growing cell cultures of YMV45 derivative strains were arrested in G2 with nocodazole and transferred to YEPRG in the presence of nocodazole at time zero to induce HO. Southern blot analysis with a *LEU2* probe of KpnI-digested genomic DNA. (C) Densitometric analysis of the product band signals (see Materials and Methods). Plotted values are the mean value with error bars denoting s.d. (*n* = 3). (D) The assay was done as in (B). * indicates a Rad51-dependent recombination product.
(TIF)

S2 Fig Mre11 protein level. Western blot with anti-Myc antibodies of extracts used for the ChIP analysis shown in Fig 8A and 8B. The same amount of protein extracts was separated on a SDS-PAGE and stained with Coomassie Blue (loading control).
(TIF)

S1 Table List of yeast strains used in this work. (DOC)

## Abbreviations

ATP: adenosine triphosphate
BSA: bovine serum albumin
ChIP: chromatin immunoprecipitation
CO: crossover
CPT: camptothecin
DDR: DNA damage response
DSB: double-strand break
dsDNA: double-stranded DNA
ssDNA: single-stranded DNA
DTT: dithiothreitol
EDTA: ethylenediaminetetraacetic acid
exp: exponentially
GFP: green fluorescent protein
HR: homologous recombination
MMS: methyl methanesulfonate
MRN: Mre11-Rad50-Nbs1
MRX: Mre11-Rad50-Xrs2
NCO: noncrossover
NHEJ: nonhomologous end joining; phleo, phleomycin
qPCR: quantitative real-time polymerase chain reaction
RFP: red fluorescent protein
SDSA: synthesis-dependent strand annealing
SSA: single-strand annealing
Tel1-kd: Tel1 kinase defective
YEPD: yeast extract peptone dextrose
YFP: yellow fluorescent protein

## References

[1] Daley JM, Gaines WA, Kwon Y, Sung P. Regulation of DNA pairing in homologous recombination. Cold Spring Harb Perspect Biol. 2014; 6: a017954 10.1101/cshperspect.a017954 25190078 PMC4413238

[2] Ciccia A, Elledge SJ. The DNA damage response: making it safe to play with knives. Mol Cell 2010; 40: 179–204. 10.1016/j.molcel.2010.09.019 20965415 PMC2988877

[3] Gobbini E, Cesena D, Galbiati A, Lockhart A, Longhese MP. Interplays between ATM/Tel1 and ATR/Mec1 in sensing and signaling DNA double-strand breaks. DNA Repair 2013; 12: 791–799. 10.1016/j.dnarep.2013.07.009 23953933

[4] Nakada D, Matsumoto K, Sugimoto K. ATM-related Tel1 associates with double-strand breaks through an Xrs2-dependent mechanism. Genes Dev. 2003; 17: 1957–1962. 12923051 10.1101/gad.1099003 PMC196250

[5] Falck J, Coates J, Jackson SP. Conserved modes of recruitment of ATM, ATR and DNA-PKcs to sites of DNA damage. Nature 2005; 434: 605–611. 15758953 10.1038/nature03442

[6] Lee JH, Paull TT. ATM activation by DNA double-strand breaks through the Mre11-Rad50-Nbs1 complex. Science 2005; 308: 551–554. 15790808 10.1126/science.1108297

[7] You Z, Chahwan C, Bailis J, Hunter T, Russell P. ATM activation and its recruitment to damaged DNA require binding to the C terminus of Nbs1. Mol Cell Biol. 2005; 25: 5363–5379. 15964794 10.1128/MCB.25.13.5363-5379.2005 PMC1156989

[8] Fukunaga K, Kwon Y, Sung P, Sugimoto K. Activation of protein kinase Tel1 through recognition of protein-bound DNA ends. Mol Cell Biol. 2011; 31: 1959–1971. 10.1128/MCB.05157-11 21402778 PMC3133365

[9] Stracker TH, Petrini JH. The MRE11 complex: starting from the ends. Nat Rev Mol Cell Biol. 2011; 12: 90–103. 10.1038/nrm3047 21252998 PMC3905242

[10] de Jager M, van Noort J, van Gent DC, Dekker C, Kanaar R, Wyman C. Human Rad50/Mre11 is a flexible complex that can tether DNA ends. Mol Cell 2001; 8: 1129–1135. 11741547 10.1016/s1097-2765(01)00381-1

[11] Hopfner KP, Karcher A, Shin DS, Craig L, Arthur LM, Carney JP, et al Structural biology of Rad50 ATPase: ATP-driven conformational control in DNA double-strand break repair and the ABC-ATPase superfamily. Cell 2000; 101: 789–800. 10892749 10.1016/s0092-8674(00)80890-9

[12] Moreno-Herrero F, de Jager M, Dekker NH, Kanaar R, Wyman C, Dekker C. Mesoscale conformational changes in the DNA-repair complex Rad50/Mre11/Nbs1 upon binding DNA. Nature 2005; 437: 440–443. 16163361 10.1038/nature03927

[13] Lammens K, Bemeleit DJ, Möckel C, Clausing E, Schele A, Hartung S, et al The Mre11:Rad50 structure shows an ATP-dependent molecular clamp in DNA double-strand break repair. Cell 2011; 145: 54–66. 10.1016/j.cell.2011.02.038 21458667 PMC3071652

[14] Hopfner KP, Craig L, Moncalian G, Zinkel RA, Usui T, Owen BA, et al The Rad50 zinc-hook is a structure joining Mre11 complexes in DNA recombination and repair. Nature 2002; 418: 562–566. 12152085 10.1038/nature00922

[15] Wiltzius JJ, Hohl M, Fleming JC, Petrini JH. The Rad50 hook domain is a critical determinant of Mre11 complex functions. Nat Struct Mol Biol. 2005; 12: 403–407. 15852023 10.1038/nsmb928

[16] Paull TT, Gellert M. The 3' to 5' exonuclease activity of Mre11 facilitates repair of DNA double-strand breaks. Mol Cell 1998; 1: 969–979. 9651580 10.1016/s1097-2765(00)80097-0

[17] Trujillo KM, Yuan SS, Lee EY, Sung P. Nuclease activities in a complex of human recombination and DNA repair factors Rad50, Mre11, and p95. J Biol Chem. 1998; 273: 21447–21450. 9705271 10.1074/jbc.273.34.21447

[18] Trujillo KM, Sung P. DNA structure-specific nuclease activities in the *Saccharomyces cerevisiae* Rad50-Mre11 complex. J Biol Chem. 2001; 276: 35458–35464. 11454871 10.1074/jbc.M105482200

[19] Williams RS, Moncalian G, Williams JS, Yamada Y, Limbo O, Shin DS, et al Mre11 dimers coordinate DNA end bridging and nuclease processing in double-strand-break repair. Cell 2008; 135:97–109. 10.1016/j.cell.2008.08.017 18854158 PMC2681233

[20] Mimitou EP, Symington LS. Sae2, Exo1 and Sgs1 collaborate in DNA double-strand break processing. Nature 2008; 455: 770–774. 10.1038/nature07312 18806779 PMC3818707

[21] Zhu Z, Chung WH, Shim EY, Lee SE, Ira G. Sgs1 helicase and two nucleases Dna2 and Exo1 resect DNA double-strand break ends. Cell 2008; 134: 981–994. 10.1016/j.cell.2008.08.037 18805091 PMC2662516

[22] Cejka P, Cannavo E, Polaczek P, Masuda-Sasa T, Pokharel S, Campbell JL, et al DNA end resection by Dna2-Sgs1-RPA and its stimulation by Top3-Rmi1 and Mre11-Rad50-Xrs2. Nature 2010; 467: 112–116. 10.1038/nature09355 20811461 PMC3089589

[23] Niu H, Chung WH, Zhu Z, Kwon Y, Zhao W, Chi P, et al Mechanism of the ATP-dependent DNA end-resection machinery from *Saccharomyces cerevisiae* . Nature 2010; 467: 108–111. 10.1038/nature09318 20811460 PMC2955862

[24] Shibata A, Moiani D, Arvai AS, Perry J, Harding SM, Genois MM, et al DNA double-strand break repair pathway choice is directed by distinct MRE11 nuclease activities. Mol Cell 2014; 53:7–18. 10.1016/j.molcel.2013.11.003 24316220 PMC3909494

[25] Paull TT, Gellert M. Nbs1 potentiates ATP-driven DNA unwinding and endonuclease cleavage by the Mre11/Rad50 complex. Genes Dev. 1999; 13: 1276–1288. 10346816 10.1101/gad.13.10.1276 PMC316715

[26] Moncalian G, Lengsfeld B, Bhaskara V, Hopfner KP, Karcher A, Alden E, et al The Rad50 signature motif: essential to ATP binding and biological function. J Mol Biol. 2004; 335: 937–951. 14698290 10.1016/j.jmb.2003.11.026

[27] Chen L, Trujillo KM, Van Komen S, Roh DH, Krejci L, Lewis LK, et al Effect of amino acid substitutions in the Rad50 ATP binding domain on DNA double strand break repair in yeast. J Biol Chem. 2005; 280: 2620–2627. 15546877 10.1074/jbc.M410192200

[28] Williams GJ, Williams RS, Williams JS, Moncalian G, Arvai AS, Limbo O, et al ABC ATPase signature helices in Rad50 link nucleotide state to Mre11 interface for DNA repair. Nat Struct Mol Biol. 2011; 18: 423–431. 10.1038/nsmb.2038 21441914 PMC3118400

[29] Deshpande RA, Williams GJ, Limbo O, Williams RS, Kuhnlein J, Lee JH, et al ATP-driven Rad50 conformations regulate DNA tethering, end resection, and ATM checkpoint signaling. EMBO J. 2014; 33: 482–500. 10.1002/embj.201386100 24493214 PMC3989629

[30] Möckel C, Lammens K, Schele A, Hopfner KP. ATP driven structural changes of the bacterial Mre11:Rad50 catalytic head complex. Nucleic Acids Res. 2012; 40: 914–927. 10.1093/nar/gkr749 21937514 PMC3258140

[31] Lim HS, Kim JS, Park YB, Gwon GH, Cho Y. Crystal structure of the Mre11-Rad50-ATPγS complex: understanding the interplay between Mre11 and Rad50. Genes Dev. 2011; 25: 1091–1104. 10.1101/gad.2037811 21511873 PMC3093124

[32] Lee JH, Mand MR, Deshpande RA, Kinoshita E, Yang SH, Wyman C, et al Ataxia telangiectasia-mutated (ATM) kinase activity is regulated by ATP-driven conformational changes in the Mre11/Rad50/Nbs1 (MRN) complex. J Biol Chem. 2013; 288: 12840–12851. 10.1074/jbc.M113.460378 23525106 PMC3642328

[33] Ritchie KB, Petes TD. The Mre11p/Rad50p/Xrs2p complex and the Tel1p function in a single pathway for telomere maintenance in yeast. Genetics 2000; 155: 475–479. 10790418 10.1093/genetics/155.1.475 PMC1461057

[34] Tsukamoto Y, Taggart AK, Zakian VA. The role of the Mre11-Rad50-Xrs2 complex in telomerase- mediated lengthening of *Saccharomyces cerevisiae* telomeres. Curr Biol. 2001; 11: 1328–1335. 11553325 10.1016/s0960-9822(01)00372-4

[35] Hirano Y, Fukunaga K, Sugimoto K. Rif1 and Rif2 inhibit localization of Tel1 to DNA ends. Mol Cell 2009; 33: 312–322. 10.1016/j.molcel.2008.12.027 19217405 PMC2662776

[36] Martina M, Clerici M, Baldo V, Bonetti D, Lucchini G, Longhese MP. A balance between Tel1 and Rif2 activities regulates nucleolytic processing and elongation at telomeres. Mol Cell Biol. 2012; 32: 1604–1617. 10.1128/MCB.06547-11 22354991 PMC3347235

[37] Wotton D, Shore D. A novel Rap1p-interacting factor, Rif2p, cooperates with Rif1p to regulate telomere length in *Saccharomyces cerevisiae* . Genes Dev. 1997; 11: 748–760. 9087429 10.1101/gad.11.6.748

[38] Mantiero D, Clerici M, Lucchini G, Longhese MP. Dual role for *Saccharomyces cerevisiae* Tel1 in the checkpoint response to double-strand breaks. EMBO Rep. 2007; 8: 380–387. 17347674 10.1038/sj.embor.7400911 PMC1852765

[39] Mallory JC, Petes TD. Protein kinase activity of Tel1p and Mec1p, two *Saccharomyces cerevisiae* proteins related to the human ATM protein kinase. Proc Natl Acad Sci USA 2000; 97: 13749–13754. 11095737 10.1073/pnas.250475697 PMC17647

[40] Zaitseva J, Jenewein S, Jumpertz T, Holland IB, Schmitt L. H662 is the linchpin of ATP hydrolysis in the nucleotide-binding domain of the ABC transporter HlyB. EMBO J. 2005; 24: 1901–1910. 15889153 10.1038/sj.emboj.7600657 PMC1142601

[41] Trujillo KM, Roh DH, Chen L, Van Komen S, Tomkinson A, Sung P. Yeast Xrs2 binds DNA and helps target Rad50 and Mre11 to DNA ends. J Biol Chem. 2003; 278: 48957–48964. 14522986 10.1074/jbc.M309877200

[42] Lee SE, Moore JK, Holmes A, Umezu K, Kolodner RD, Haber JE. *Saccharomyces* Ku70, Mre11/Rad50 and RPA proteins regulate adaptation to G2/M arrest after DNA damage. Cell 1998; 94: 399–409. 9708741 10.1016/s0092-8674(00)81482-8

[43] Kaye JA, Melo JA, Cheung SK, Vaze MB, Haber JE, Toczyski DP. DNA breaks promote genomic instability by impeding proper chromosome segregation. Curr Biol. 2004; 14: 2096–2106. 15589151 10.1016/j.cub.2004.10.051

[44] Lobachev K, Vitriol E, Stemple J, Resnick MA, Bloom K. Chromosome fragmentation after induction of a double-strand break is an active process prevented by the RMX repair complex. Curr Biol. 2004; 14: 2107–2112. 15589152 10.1016/j.cub.2004.11.051

[45] Lee K, Zhang Y, Lee SE. *Saccharomyces cerevisiae* ATM orthologue suppresses break-induced chromosome translocations. Nature 2008; 454: 543–546. 10.1038/nature07054 18650924

[46] Nakai W, Westmoreland J, Yeh E, Bloom K, Resnick MA. Chromosome integrity at a double-strand break requires exonuclease 1 and MRX. DNA Repair 2011; 10: 102–110. 10.1016/j.dnarep.2010.10.004 21115410 PMC3031249

[47] Cannavo E, Cejka P. Sae2 promotes dsDNA endonuclease activity within Mre11-Rad50-Xrs2 to resect DNA breaks. Nature 2014; 514: 122–125. 10.1038/nature13771 25231868

[48] Zierhut C, Diffley JF. Break dosage, cell cycle stage and DNA replication influence DNA double strand break response. EMBO J 2008; 27: 1875–1885. 10.1038/emboj.2008.111 18511906 PMC2413190

[49] Tsubouchi H, Ogawa H. Exo1 roles for repair of DNA double-strand breaks and meiotic crossing over in *Saccharomyces cerevisiae* . Mol Biol Cell 2000; 11: 2221–2233. 10888664 10.1091/mbc.11.7.2221 PMC14915

[50] Tittel-Elmer M, Alabert C, Pasero P, Cobb JA. The MRX complex stabilizes the replisome independently of the S phase checkpoint during replication stress. EMBO J. 2009; 28: 1142–1156. 10.1038/emboj.2009.60 19279665 PMC2683708

[51] Lisby M, Antúnez de Mayolo A, Mortensen UH, Rothstein R. Cell cycle-regulated centers of DNA double-strand break repair. Cell Cycle 2003; 2: 479–483. 12963848

[52] Clerici M, Mantiero D, Lucchini G, Longhese MP. The *Saccharomyces cerevisiae* Sae2 protein promotes resection and bridging of double strand break ends. J Biol Chem. 2005; 280: 38631–38638. 16162495 10.1074/jbc.M508339200

[53] Mehta A, Haber JE. Sources of DNA double-strand breaks and models of recombinational DNA repair. Cold Spring Harb Perspect Biol. 2014; 6:a016428 10.1101/cshperspect.a016428 25104768 PMC4142968

[54] Vaze MB, Pellicioli A, Lee SE, Ira G, Liberi G, Arbel-Eden A, et al Recovery from checkpoint-mediated arrest after repair of a double-strand break requires Srs2 helicase. Mol Cell 2002; 10: 373–385. 12191482 10.1016/s1097-2765(02)00593-2

[55] Ivanov EL, Sugawara N, Fishman-Lobell J, Haber JE. Genetic requirements for the single-strand annealing pathway of double-strand break repair in *Saccharomyces cerevisiae* . Genetics 1996; 142: 693–704. 8849880 10.1093/genetics/142.3.693 PMC1207011

[56] Strathern JN, Klar AJ, Hicks JB, Abraham JA, Ivy JM, Nasmyth KA, et al Homothallic switching of yeast mating type cassettes is initiated by a double-stranded cut in the *MAT* locus. Cell 1982; 31: 183–192. 6297747 10.1016/0092-8674(82)90418-4

[57] Nassif N, Penney J, Pal S, Engels WR, Gloor GB. Efficient copying of nonhomologous sequences from ectopic sites via P-element-induced gap repair. Mol Cell Biol. 1994; 14: 1613–1625. 8114699 10.1128/mcb.14.3.1613-1625.1994 PMC358520

[58] Ferguson DO, Holloman WK. Recombinational repair of gaps in DNA is asymmetric in *Ustilago maydis* and can be explained by a migrating D-loop model. Proc Natl Acad Sci USA 1996; 93: 5419–5424. 8643590 10.1073/pnas.93.11.5419 PMC39261

[59] Saponaro M, Callahan D, Zheng X, Krejci L, Haber JE, Klein HL et al Cdk1 targets Srs2 to complete synthesis-dependent strand annealing and to promote recombinational repair. PLoS Genet. 2010; 6: e1000858 10.1371/journal.pgen.1000858 20195513 PMC2829061

[60] Haber JE. Mating-type genes and *MAT* switching in *Saccharomyces cerevisiae* . Genetics 2012; 191: 33–64. 10.1534/genetics.111.134577 22555442 PMC3338269

[61] Aylon Y, Liefshitz B, Kupiec M. The CDK regulates repair of double-strand breaks by homologous recombination during the cell cycle. EMBO J. 2004; 23: 4868–4875. 15549137 10.1038/sj.emboj.7600469 PMC535085

[62] Ira G, Pellicioli A, Balijja A, Wang X, Fiorani S, Carotenuto W, et al DNA end resection, homologous recombination and DNA damage checkpoint activation require CDK1. Nature 2004; 431: 1011–1017. 15496928 10.1038/nature02964 PMC4493751

[63] Lee SE, Pâques F, Sylvan J, Haber JE. Role of yeast SIR genes and mating type in directing DNA double-strand breaks to homologous and non-homologous repair paths. Curr Biol. 1999; 9: 767–770. 10421582 10.1016/s0960-9822(99)80339-x

[64] Steininger S, Gomez-Paramio I, Braselmann H, Fellerhoff B, Dittberner D, Eckardt-Schupp F, Moertl S. Xrs2 facilitates crossovers during DNA double-strand gap repair in yeast. DNA Repair 2008; 7:1563–1577. 10.1016/j.dnarep.2008.06.004 18599383

[65] Clerici M, Mantiero D, Lucchini G, Longhese MP. The *Saccharomyces cerevisiae* Sae2 protein negatively regulates DNA damage checkpoint signalling. EMBO Rep. 2006; 7: 212–218. 16374511 10.1038/sj.embor.7400593 PMC1369247

[66] Usui T, Ogawa H, Petrini JH. A DNA damage response pathway controlled by Tel1 and the Mre11 complex. Mol Cell; 2001 7: 1255–1266. 11430828 10.1016/s1097-2765(01)00270-2

[67] McGee JS, Phillips JA, Chan A, Sabourin M, Paeschke K, Zakian VA. Reduced Rif2 and lack of Mec1 target short telomeres for elongation rather than double-strand break repair. Nat Struct Mol Biol. 2010; 17: 1438–1445. 10.1038/nsmb.1947 21057524 PMC3058685

[68] Marcand S, Pardo B, Gratias A, Cahun S, Callebaut I. Multiple pathways inhibit NHEJ at telomeres. Genes Dev. 2008; 22: 1153–1158. 10.1101/gad.455108 18451106 PMC2335312

[69] Cremona CA, Behrens A. ATM signalling and cancer. Oncogene 2014; 33: 3351–3360. 10.1038/onc.2013.275 23851492

[70] Al-Ahmadie H, Iyer G, Hohl M, Asthana S, Inagaki A, Schultz N, et al Synthetic lethality in ATM-deficient RAD50-mutant tumors underlies outlier response to cancer therapy. Cancer Discov. 2014; 4: 1014–1021. 10.1158/2159-8290.CD-14-0380 24934408 PMC4155059

[71] Bonetti D, Anbalagan S, Lucchini G, Clerici M, Longhese MP. Tbf1 and Vid22 promote resection and non-homologous end joining of DNA double-strand break ends. EMBO J. 2013; 32: 275–289. 10.1038/emboj.2012.327 23222485 PMC3553383

[72] Trovesi C, Falcettoni M, Lucchini G, Clerici M, Longhese MP. Distinct Cdk1 requirements during single-strand annealing, noncrossover, and crossover recombination. PLoS Genet. 2011; 7:e1002263 10.1371/journal.pgen.1002263 21901114 PMC3161966

[73] Viscardi V, Bonetti D, Cartagena-Lirola H, Lucchini G, Longhese MP. MRX-dependent DNA damage response to short telomeres. Mol Biol Cell. 2007; 18: 3047–3058. 17538011 10.1091/mbc.E07-03-0285 PMC1949382

[74] Chen L, Trujillo K, Ramos W, Sung P, Tomkinson AE. Promotion of Dnl4-catalyzed DNA end-joining by the Rad50/Mre11/Xrs2 and Hdf1/Hdf2 complexes. Mol Cell 2001; 8: 1105–1115. 11741545 10.1016/s1097-2765(01)00388-4

