# Supplementary figures and images for: Tel1 and Rif2 Regulate MRX Functions in End-Tethering and Repair of DNA Double-Strand Breaks
Source: PLoS Biol. 2016 Feb 22;14(2):e1002387. doi: 10.1371/journal.pbio.1002387 (PMC4762649; doi:10.1371/journal.pbio.1002387)

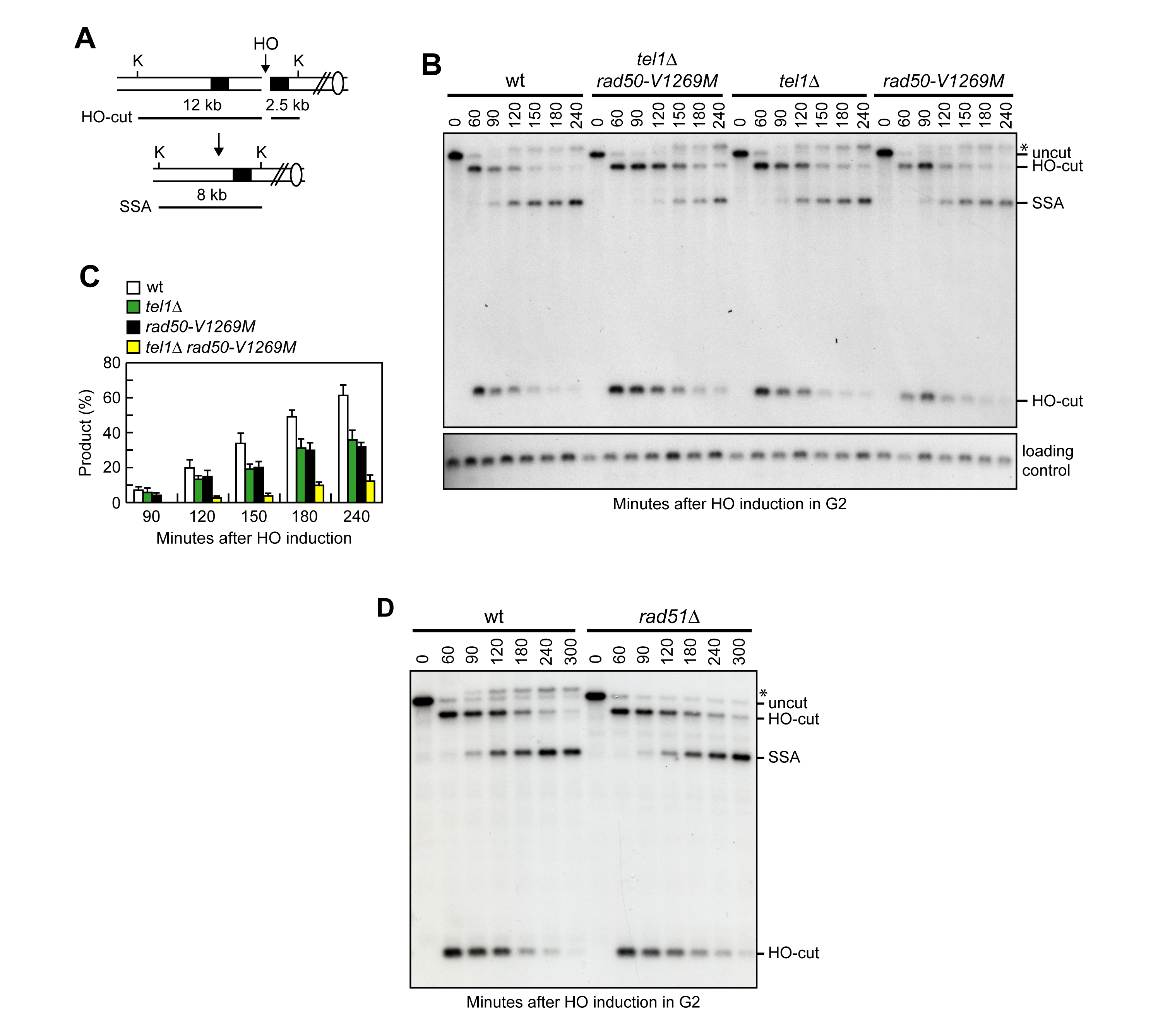

Supplement: S1 Fig — (A) Map of the YMV45 chromosome III region where the HO-cut site is flanked by homologous leu2 sequences (black boxes) that are 4.6 kb apart. HO-induced DSB formation results in generation of 12 kb and 2.5 kb DNA fragments (HO-cut) that can be detected by Southern blot analysis of KpnI-digested genomic DNA with a LEU2 probe. DSB repair by SSA generates a product of 8 kb (SSA). K, KpnI. (B) DSB repair by SSA. YEPR exponentially growing cell cultures of YMV45 derivative strains were arrested in G2 with nocodazole and transferred to YEPRG in the presence of nocodazole at time zero to induce HO. Southern blot analysis with a LEU2 probe of KpnI-digested genomic DNA. (C) Densitometric analysis of the product band signals (see Materials and Methods). Plotted values are the mean value with error bars denoting s.d. (n = 3). (D) The assay was done as in (B). * indicates a Rad51-dependent recombination product. (TIF) [file pbio.1002387.s002.tif]

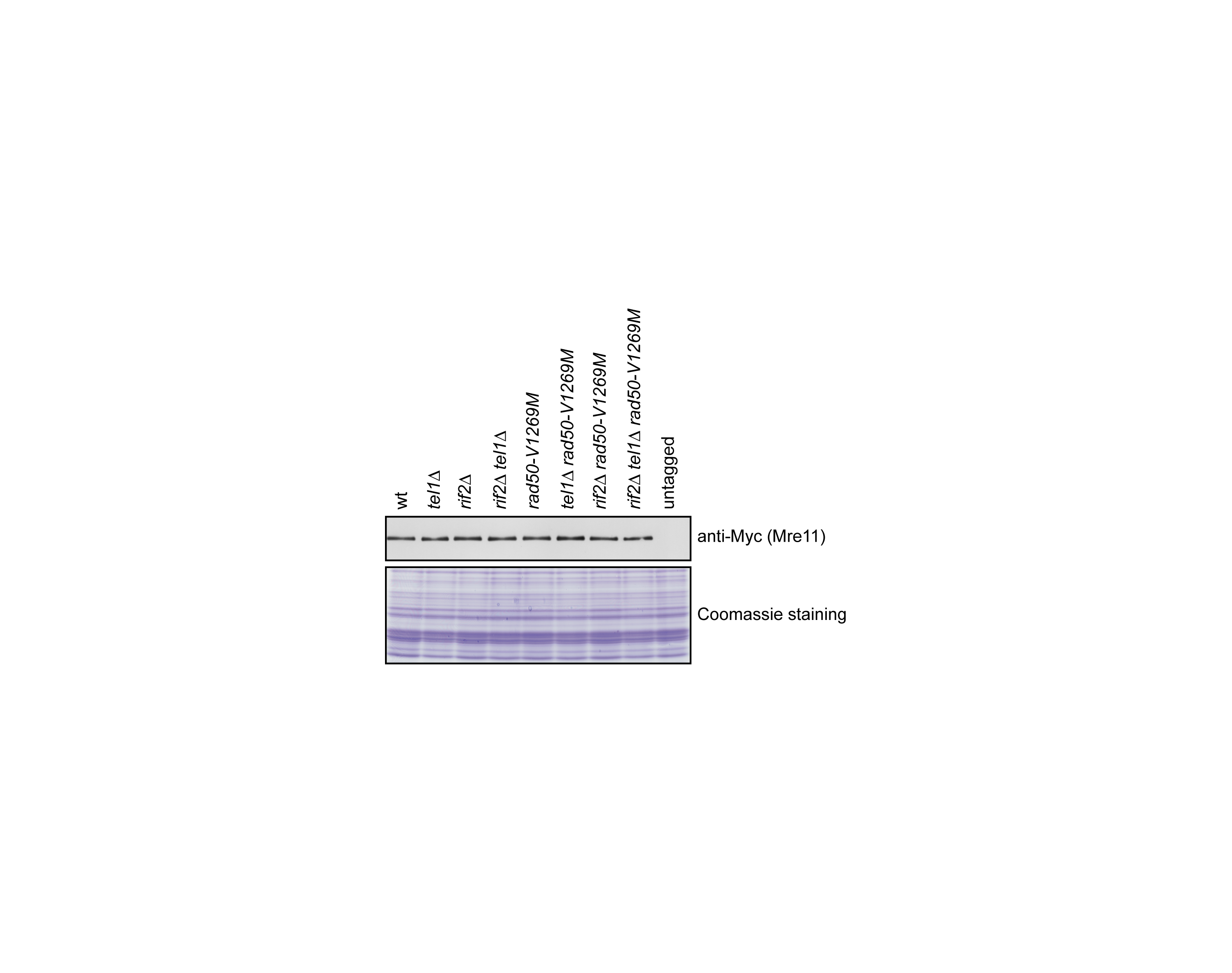

Supplement: S2 Fig — Western blot with anti-Myc antibodies of extracts used for the ChIP analysis shown in Fig 8A and 8B. The same amount of protein extracts was separated on a SDS-PAGE and stained with Coomassie Blue (loading control). (TIF) [file pbio.1002387.s003.tif]
