# Supplementary material for: Tel1 and Rif2 Regulate MRX Functions in End-Tethering and Repair of DNA Double-Strand Breaks
Source: PLoS Biol. 2016 Feb 22;14(2):e1002387. doi: 10.1371/journal.pbio.1002387 (PMC4762649; doi:10.1371/journal.pbio.1002387)
Supplement: S1 Table — (DOC) [file pbio.1002387.s004.doc]

| Strain | Relevant genotype | Source |
| --- | --- | --- |
| W303 | *MAT*a/α *ade2-1 can1-100 his3-11,15 leu2-3,112 trp1-1 ura3-1 rad5-535* | 1 |
| DMP5781/1B | W303 *MAT*a *rad50-V1269M::KANMX* | This study |
| DMP3335/2A | W303 *MAT*α *tel1∆::HIS3* | This study |
| DMP5928/2C | W303 *MAT*a *rad50-V1269M::KANMX* *tel1∆::HIS3* | This study |
| DMP5928/4A | W303 *MAT*α *rad50-V1269M::KANMX* *tel1∆::HIS3* | This study |
| DMP4267/3B | W303 *MAT*α *rad50∆::HIS3* | This study |
| YLL3575.1 | W303 *MAT*a *tel1-kd::LEU2* | This study |
| DMP6167/2B | W303 *MAT*a *rad50-V1269M::KANMX* *tel1-kd::LEU2* | This study |
| YLL1134.1 | W303 *MAT*a *rif2∆::KANMX* | This study |
| DMP6121/2D | W303 *MAT*a *rif2∆::KANMX rad50-V1269M::KANMX* | This study |
| DMP6121/5B | W303 *MAT*a *rif2∆::KANMX rad50-V1269M::KANMX* | This study |
| DMP6121/10A | W303 *MAT*a *rif2∆::KANMX tel1∆::HIS3* | This study |
| DMP6121/6A | W303 *MAT*a *rif2∆::KANMX tel1∆::HIS3 rad50-V1269M::KANMX* | This study |
| DMP6121/5D | W303 *MAT*a *rif2∆::KANMX tel1∆::HIS3 rad50-V1269M::KANMX* | This study |
| YLL2479.1 | W303 *MAT*a *dnl4∆::KANMX* | 2 |
| DMP6176/7B | W303 *MAT*a *rad50-V1269M::KANMX xrs2-11::KANMX* | This study |
| DMP6311/27C | W303 *MAT*a *rad50-V1269M::KANMX xrs2-11::KANMX rif2∆::HIS3* | This study |
| JKM139 | *MAT*a *hml∆*::*ADE1,* *hmr∆::ADE1*, *ade1-100, lys5,*  *leu2-3,112, trp1::hisG ura3-52, ho, ade3::GAL-HO site* | 3 |
| YLL3501.1 | JKM139 *MAT*a *RAD50-3HA::URA3* | This study |
| DMP6046/7A | JKM139 *MAT*a *rad50-V1269M-3HA::URA3* | This study |
| YLL1854.2 | JKM139 *MAT*a *MRE11-18MYC::TRP1* | This study |
| DMP6021/4A | JKM139 *MAT*a *MRE11-18MYC::TRP1* *rad50-V1269M::KANMX* | This study |
| YLL3222.6 | JKM139 *MAT*a *TEL1-3HA::NATMX* | This study |
| DMP5841/4C | JKM139 *MAT*a *TEL1-3HA::NATMX* *rad50-V1269M::KANMX* | This study |
| DMP5819/3D | JKM139 *MAT*a *rad50-V1269M::KANMX* | This study |
| YLL1794.3 | JKM139 *MAT*a *tel1∆::NATMX* | This study |
| DMP6312/1A | JKM139 *MAT*a *tel1-kd::LEU2 RAD50-3HA::URA3* | This study |
| DMP6209/6C | JKM139 *MAT*a *tel1-kd::LEU2 MRE11-18MYC::TRP1* | This study |
| DMP5960/1A | JKM139 *MAT*a *rad50-V1269M::KANMX tel1∆::NATMX* | This study |
| DMP6094/6A | JKM139 *MAT*a *RAD50-3HA::URA3 tel1∆::NATMX* | This study |
| DMP6214/1D | JKM139 *MAT*a *MRE11-18MYC::TRP1 tel1∆::NATMX* | This study |
| DMP6095/4A | JKM139 *MAT*a *rad50-V1269M-3HA::URA3 tel1∆::NATMX* | This study |
| DMP6224/11D | JKM139 *MAT*α *MRE11-18MYC::TRP1 rad50-V1269M::KANMX tel1∆::NATMX* | This study |
| DMP6211/5B | JKM139 *MATa MRE11-18MYC::TRP1 rif2∆::HPHMX* | This study |
| DMP6214/3B | JKM139 *MAT*a *MRE11-18MYC::TRP1 rif2∆::HPHMX tel1∆::NATMX* | This study |
| DMP6211/2D | JKM139 *MAT*a *MRE11-18MYC::TRP1 rad50-V1269M::KANMX rif2∆::HPHMX* | This study |
| DMP6224/1C | JKM139 *MAT*a *MRE11-18MYC::TRP1 rad50-V1269M::KANMX rif2∆::HPHMX tel1∆::NATMX* | This study |
| YLL3611.1 | JKM139 *MATa RIF2-18MYC::TRP1* | This study |
| YLL3611.1 | JKM139 *MATa RIF2-18MYC::TRP1 mre11∆::HPHMX* | This study |
| YLL3613.2 | JKM139 *MAT*a *RIF2-18MYC::TRP1 rad50-V1269M::KANMX* | This study |
| DMP6098/4A | JKM139 *MAT*a *MRE11-18MYC::TRP1 RAD50-3HA::URA3* | This study |
| DMP6096/1C | JKM139 *MAT*a *MRE11-18MYC::TRP1 rad50-V1269M-3HA::URA3* | This study |
| YJK40.6 | *MAT*Δ *hml*Δ *hmr*Δ *can1 lys5 ade2 leu2 trp1 ura3 his3 ade3*::*GAL-HO VII*::*TRP1-HO LacI-GFP*::*URA3 LacO*::*LYS5 LacO*::*KanR* | 4 |
| YLL3644.6 | YJK40.6 *rif2∆::LEU2* | This study |
| YLL3617.2 | YJK40.6 *tel1∆::NATMX* | This study |
| YLL3643.2 | YJK40.6 *tel1∆::NATMX rif2∆::LEU2* | This study |
| YLL3641.6 | YJK40.6 *rad50-V1269M::HPHMX* | This study |
| YLL3642.1 | YJK40.6 *rad50-V1269M::HPHMX rif2∆::LEU2* | This study |
| YLL3623.12 | YJK40.6 *rad50-V1269M::HPHMX tel1∆::NATMX* | This study |
| YLL3653.5 | YJK40.6 *rad50-V1269M::HPHMX tel1∆::NATMX rif2∆::LEU2* | This study |
| YLL1731.29 | YJK40.6 *mre11∆::NATMX* | 5 |
| YMV45 | *ho hml::ADE1 mata::hisG hmr::ADE1 leu2::leu2(Asp718-SalI)-URA3-*pBR332*-MATa ade3::GAL::HO ade1 lys5 ura3-52 trp1::hisG* | 6 |
| YLL3648.16 | YMV45 *rad50-V1269M::HPHMX* | This study |
| YLL3647.2 | YMV45 *rad50-V1269M::HPHMX tel1∆::KANMX* | This study |
| YLL3529.1 | YMV45 *tel1∆::KANMX* | This study |
| tGI354 | *ho hml∆::ADE1 MATa-inc hmr∆::ADE1 ade1 leu2-3;112 lys5 trp1::hisG ura3-52 ade3::GAL::HO arg5,6::MATa::HPHMX* | 7 |
| YLL3678.12 | tGI354 *tel1∆::NATMX* | This study |
| YLL3701.44 | tGI354 *rad50-V1269M::KANMX* | This study |
| YLL3686.4 | tGI354 *tel1∆::NATMX rad50-V1269M::KANMX* | This study |
| W4441-11C | *MAT::HIS3  bar1::LEU2 trp1-1 his3-11,15::YFP-LacI-his3-x leu2-3,112::LacO-LEU2 HO-iYCL018W(leu2-3,112) TetR-mRFP1 URA3::tetOx224* | 8 |
| YLL3698.42 | W4441-11C *tel1∆::NATMX* | This study |
| YLL3700.23 | W4441-11C *rad50-V1269M::KANMX* | This study |
| YLL3699.2 | W4441-11C *tel1∆::NATMX rad50-V1269M::KANMX* | This study |

**References**

1. Bonetti D, Martina M, Clerici M, Lucchini G and Longhese MP. Multiple pathways regulate 3' overhang generation at *S. cerevisiae* telomeres. Mol Cell. 2009; 35: 70-81.

2. [Bonetti D](http://www.ncbi.nlm.nih.gov/pubmed/?term=Bonetti D%5BAuthor%5D&cauthor=true&cauthor_uid=23222485), [Anbalagan S](http://www.ncbi.nlm.nih.gov/pubmed/?term=Anbalagan S%5BAuthor%5D&cauthor=true&cauthor_uid=23222485), [Lucchini G](http://www.ncbi.nlm.nih.gov/pubmed/?term=Lucchini G%5BAuthor%5D&cauthor=true&cauthor_uid=23222485), [Clerici M](http://www.ncbi.nlm.nih.gov/pubmed/?term=Clerici M%5BAuthor%5D&cauthor=true&cauthor_uid=23222485), [Longhese MP](http://www.ncbi.nlm.nih.gov/pubmed/?term=Longhese MP%5BAuthor%5D&cauthor=true&cauthor_uid=23222485). Tbf1 and Vid22 promote resection and non-homologous end joining of DNA double-strand break ends. EMBO J. 2013; 32: 275-289.

3. Lee SE, Moore JK, Holmes A, Umezu K, Kolodner RD, Haber JE. *Saccharomyces* Ku70, Mre11/Rad50 and RPA proteins regulate adaptation to G2/M arrest after DNA damage. Cell. 1998; 94: 399-409.

4. Kaye JA, Melo JA, Cheung SK, Vaze MB, Haber JE, Toczyski DP. [DNA breaks promote genomic instability by impeding proper chromosome segregation.](http://www.ncbi.nlm.nih.gov/pubmed/15589151) Curr Biol. 2004; 14: 2096-2106.

5. [Clerici M](http://www.ncbi.nlm.nih.gov/pubmed/?term=Clerici M%5BAuthor%5D&cauthor=true&cauthor_uid=16162495), [Mantiero D](http://www.ncbi.nlm.nih.gov/pubmed/?term=Mantiero D%5BAuthor%5D&cauthor=true&cauthor_uid=16162495), [Lucchini G](http://www.ncbi.nlm.nih.gov/pubmed/?term=Lucchini G%5BAuthor%5D&cauthor=true&cauthor_uid=16162495), [Longhese MP](http://www.ncbi.nlm.nih.gov/pubmed/?term=Longhese MP%5BAuthor%5D&cauthor=true&cauthor_uid=16162495). The *Saccharomyces cerevisiae* Sae2 protein promotes resection and bridging of double strand break ends. [J Biol Che](http://www.ncbi.nlm.nih.gov/pubmed/16162495)m. 2005; 280: 38631-38638.

6. Vaze MB, Pellicioli A, Lee SE, Ira G, Liberi G, Arbel-Eden A, Foiani M, Haber JE. Recovery from checkpoint-mediated arrest after repair of a double-strand break requires Srs2 helicase. Mol Cell. 2002; 10: 373-385.

7. Saponaro M, Callahan D, Zheng X, Krejci L, Haber JE, Klein HL, Liberi G. Cdk1 targets Srs2 to complete synthesis-dependent strand annealing and to promote recombinational repair. PLoS Genet. 2010; 6: e1000858.

8. Lisby M, Antúnez de Mayolo A, Mortensen UH, Rothstein R. [Cell cycle-regulated centers of DNA double-strand break repair.](http://www.ncbi.nlm.nih.gov/pubmed/12963848) Cell Cycle. 2003; 2: 479-483.
